# The molecular mechanisms of CD8^+^ T cell responses to SARS-CoV-2 infection mediated by TCR-pMHC interactions

**DOI:** 10.3389/fimmu.2024.1468456

**Published:** 2024-10-10

**Authors:** Shasha Deng, Zhihao Xu, Jing Hu, Yunru Yang, Fang Zhu, Zhuan Liu, Hongliang Zhang, Songquan Wu, Tengchuan Jin

**Affiliations:** ^1^ Center of Disease Immunity and Intervention, College of Medicine, Lishui University, Lishui, China; ^2^ Department of Obstetrics and Gynecology, The First Affiliated Hospital of University of Science and Technology of China (USTC), Division of Life Sciences and Medicine, University of Science and Technology of China, Hefei, Anhui, China; ^3^ Department of Experimental Radiation Oncology, The University of Texas MD Anderson Cancer Center, Houston, TX, United States; ^4^ Laboratory of Structural Immunology, the Chinese Academy of Sciences (CAS) Key Laboratory of Innate Immunity and Chronic Disease, School of Basic Medical Sciences, Division of Life Sciences and Medicine, University of Science and Technology of China, Hefei, China; ^5^ Institute of Health and Medicine, Hefei Comprehensive National Science Center, Hefei, Anhui, China; ^6^ Biomedical Sciences and Health Laboratory of Anhui Province, University of Science & Technology of China, Hefei, China; ^7^ Clinical Research Hospital of Chinese Academy of Sciences (Hefei), University of Science and Technology of China, Hefei, China

**Keywords:** SARS-CoV-2, cytotoxic T lymphocytes (CTL), epitope, HLA, TCR repertoire, TCR-pHLA, mutations, TCR-pMHC

## Abstract

Cytotoxic CD8^+^ T lymphocytes (CTLs) have been implicated in the severity of COVID-19. The TCR-pMHC ternary complex, formed by the T cell receptor (TCR) and peptide-MHC (major histocompatibility complex), constitutes the molecular basis of CTL responses against SARS-CoV-2. While numerous studies have been conducted on T cell immunity, the molecular mechanisms underlying CTL-mediated immunity against SARS-CoV-2 infection have not been well elaborated. In this review, we described the association between HLA variants and different immune responses to SARS-CoV-2 infection, which may lead to varying COVID-19 outcomes. We also summarized the specific TCR repertoires triggered by certain SARS-CoV-2 CTL epitopes, which might explain the variations in disease outcomes among different patients. Importantly, we have highlighted the primary strategies used by SARS-CoV-2 variants to evade T-cell killing: disrupting peptide-MHC binding, TCR recognition, and antigen processing. This review provides valuable insights into the molecule mechanism of CTL responses during SARS-CoV-2 infection, aiding efforts to control the pandemic and prepare for future challenges.

## Graphical Abstract

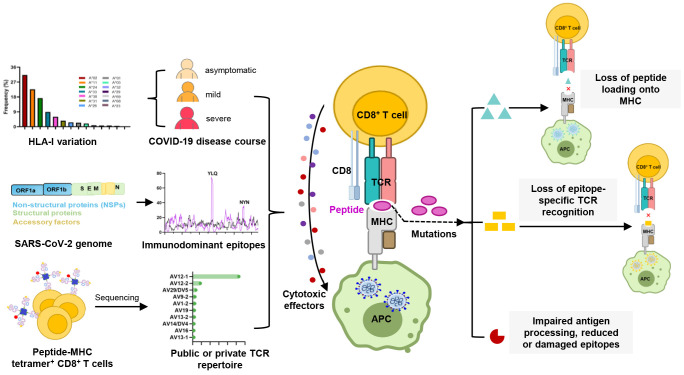

## 1 Introduction

In late 2019, the emergence of Severe Acute Respiratory Syndrome Coronavirus 2 (SARS-CoV-2) as a novel human coronavirus drew global attention to the fight against this infection (1). SARS-CoV-2 infection causes COVID-19 disease, with clinical presentations ranging from mild or asymptomatic to severe and fatal respiratory illness (2). Approximately 15% of confirmed cases are classified as severe, most occurring in individuals over 65 years of age or those with underlying medical conditions (3).

During SARS-CoV-2 infection, neutralizing antibodies, CD4^+^ helper T cells, and CD8^+^ killer T cells all contribute to controlling the virus and providing protection against viral pathogens (4, 5). Unlike the transient and heterogeneous nature of neutralizing antibodies (6–8), T cells play a critical role in conferring immune memory and establishing long-term memory responses (9–13). For closely related coronaviruses, such as SARS-CoV-1, memory T cell responses have been detected up to 17 years after infection (14, 15). Most COVID-19 convalescent patients exhibit broad and robust SARS-CoV-2-specific T cell responses (11–13, 16–20). It has been reported that functional SARS-CoV-2-specific T cell responses are retained at 6 months following infection (21, 22). Moreover, SARS-CoV-2-specific T cell responses were detectable in antibody-negative patients or in patients with B cell-deficient agammaglobulinemia, suggesting that T cells may effectively respond to the virus even in the absence or insufficiency of antibody responses (11, 16). Consistent with the positive effects observed in convalescents, the reduction of T cell lymphocytes is often used as a marker of disease severity (17, 18). Although circulating SARS-CoV-2-specific CTLs are less consistently observed than CD4^+^ T cells (4, 10, 13), their presence is generally associated with better COVID-19 outcomes (4, 12). The proportion of multifunctional CTLs is higher in mild patients compared to those with severe symptoms, further highlighting the positive role of CTLs in mitigating disease severity (12). Both *in vivo* and *in vitro* studies have demonstrated significant activation of CTLs during SARS-CoV-2 infection (10, 17, 23, 24).

The breadth and nature of the cellular immune response to viral infection are driven by the diversity of the T cell receptor (TCR) and major histocompatibility complex (MHC, HLA in humans). The tripartite interaction of TCR-peptide-MHC (TCR-pMHC) forms the basis for CTL responses against viral infections and malignancies, while maintaining auto-tolerance and averting autoimmune diseases. The antigen specificity of CTL responses is influenced by the expression of host HLA class I (HLA-I) alleles, each of which presents a virus-derived peptide ranging from 8 to 11 amino acids in length (25). Certain locations within antigenic epitopes, also described as anchor residues, have been shown to be critical for antigen presentation, and mutations in these anchor residues may disrupt peptide binding to HLA-I molecules (26, 27). Moreover, mutations within epitopes may also impact the interaction of TCRs with antigenic peptides, as seen with the P272L mutation in the YLQ (A*02/S_269-277_, YLQPRTFLL) epitope and the Y453F mutation in the NYN (A*24/S_448-456_, NYNYLYRLF) epitope (28, 29). Such mutations may interfere with the TCR-pHLA tripartite interaction, potentially leading to the failure of T cell activation, a phenomenon known as T cell immune escape (20, 29–31).

Given the importance of TCR-pMHC interaction during SARS-CoV-2 infection, there is a particular need for in-depth studies and a comprehensive understanding of TCR-pMHC complex. However, the extent to which MHC polymorphism and TCR diversity, especially concerning epitope specificity, contribute to CTL responses remains ambiguous. In this review, we evaluated the published studies about CTL immune response against SARS-CoV-2, focusing on the epitope-presenting mechanism of TCR-pHLA complexes, HLA variation in the context of various COVID-19 disease outcomes, and the characteristic of peptide-specific TCR repertoire. We also discussed how SARS-CoV-2 variants of concern evade CTL immunity and emphasized potential strategies in response to Omicron and future variants, providing a basis for vaccine optimization and prevention of reinfection.

## 2 HLA-I variation and its association with COVID-19 disease course

HLA-I genes primarily encompass the classical, highly polymorphic HLA-A, HLA-B, and HLA-C genes, as well as non-classical HLA-E, HLA-F and HLA-G with limited polymorphisms. HLA variation directly affects the binding affinities of HLA molecules to antigenic peptides, thereby influencing the recognition of pathogen-derived antigens by immune cells (32–36). To date, pathogen-driven HLA selection has been proposed and demonstrated in studies of various infectious diseases. A well-documented example of HLA alleles influence viral infections is HIV (human immunodeficiency virus) infection. Certain HLA molecules, like HLA-B*27, B*57, and B*58:01, can accommodate specific HIV antigens and trigger effective immune responses (37). The progression of HIV infection is strongly associated with the homozygosity of HLA-I genes and differential HLA expression levels (38, 39). Additionally, HLA variation has been linked to hepatitis B, hepatitis C, and several other infectious diseases (40).

Given the pandemic nature of SARS-CoV-2 and the inherent difficulties in assessing the risk of infection, the most robust genetic association studies of SARS-CoV-2 infection have mainly focused on disease outcomes. Thus far, numerous studies have explored the association between HLA alleles and COVID-19 outcomes, but without a clear consensus. In fact, some large studies, either genome-wide association studies (41) or large HLA databases (42), have failed to show significant effects of HLA alleles on disease. Different populations may possess different alleles associated with susceptibility, depending on the HLA allele pool present in each population. In addition to study design and statistical differences, this may be a reason why no conclusive association between HLA and COVID-19 has been reported to date (43–52).

Nevertheless, some correlated findings have emerged and are summarized in **Table 1**. For instance, at least two studies have reported an association between HLA-A*01:01 and diminished CTL responses (53, 54). In two other independent studies, A*11:01 and B*51:01 were identified to be associated with severe COVID-19 disease (51, 55). HLA-C*04:01 has also been found to be associated with a severe clinical course of COVID-19, with patients carrying this allele having twice the risk of requiring mechanical ventilation (56). HLA-C*14:02 allele was significantly predisposed to the worst outcomes in COVID-19 patients (51). HLA-E*01:01 allele and heterozygous HLA-E*01:01/03 genotype are associated with severe COVID-19, which may account for the individual difference in NK cell responses following SARS-CoV-2 infection (57). HLA-B*15:27, B*27:07, and C*07:29 genotypes have been found at high frequencies in infected patients (46, 48). Schindler et al. found an association between the A*30:02 allele and viral infection (58). A comprehensive computer analysis of peptide-HLA-I binding affinities across over a hundred HLA-A/B/C genotypes revealed that B*46:01 has the fewest predicted binding peptides for SARS-CoV-2 (59). This suggests that individuals with B*46:01 may be particularly susceptible to COVID-19 (59), a conclusion also supported by an earlier study related to SARS-CoV-1 (63). Moreover, some HLA genotypes have shown strong associations with mild disease and cross-reactive CTL responses. A strong association was reported between B*15:01 and asymptomatic infection in patients capable of clearing the virus during the early stages of infection (60, 61). Predicted SARS-CoV-2 peptides presented by B*15:03 are highly conserved across all other human coronaviruses (HCoVs), implying the potential for cross-protective T cell immunity (59). HLA-A*02:05, B*58:01, and C*07:01 have been reported to be associated with protective effect against SARS-CoV-2 infection. Overall, despite mixed results, some consistent patterns are beginning to emerge in the association between HLA alleles and SARS-CoV-2 infection. These patterns may offer a valuable foundation for interpreting studies related to viral antigen presentation.

**Table 1:** Summary of HLA associations with COVID-19 outcomes.

HLA-I allele	Association with	Country	OR (95% CI)	*p*-value	Reference
A*01:01	Severe disease	Italy	*	*	(53, 54)
A*11:01	Severe disease	Japan	3.41 (1.50-7.73)	3.34E-03	(55)
	Severe disease	China	2.33	8.51E-03	(51)
B*51:01	Severe disease	China	3.38	0.007017	(51)
C*04:01	Severe disease	Germany	5.4 (1.9-15.1)	1.10E-04	(56)
C*14:02	Severe disease	China	4.75	3.03E-03	(51)
E*01:01/03	Severe disease	China	*	*	(57)
B*15:27	High frequencies in patients	China	3.6	0.001	(46)
B*27:07	High frequencies in patients	Italy	*	0.00001	(48)
C*07:29	High frequencies in patients	China	130.20	0.001	(46)
A*30:02	Viral infection	USA	2.2 (1.4-3.6)	1.70E-03	(58)
B*46:01	Weak binding peptides	*	*	*	(59)
B*15:01	Asymptomatic infection	USA	2.4 (1.54-3.64)	5.67E-05	(60, 61)
B*15:03	Cross-protective immunity	*	*	*	(59)
A*02:05	Protective effect	Italy	0.1 (0-0.6)	0.015	(62)
B*58:01	Protective effect	Italy	0.1 (0-0.6)	0.015	(62)
C*07:01	Protective effect	Italy	0.1 (0-0.6)	0.015	(62)

OR, odds ratio; *, not available.

## 3 Analysis of SARS-CoV-2 CTL epitope distribution and immunodominance

SARS-CoV-2 epitopes have been identified for over 30 HLA-I alleles, such as HLA-A*02:01, A*24:02, A*01:01, and B*07:02 (64). According to the information provided by Immune Epitope Database (IEDB, http://tools.iedb.org/immunomebrowser/) (65), CTL responses are directed at multi-antigen, encompassing structural proteins such as S (spike), N (nucleoprotein), and M (membrane protein), as well as non-structural proteins like ORF3a, ORF7a and ORF8. Viral proteins that are abundantly expressed in SARS-CoV-2-infected cells tend to be the most dominant targets in the T cell response to viral invasion (10, 11, 13, 66, 67). This phenomenon may be attributed to the fact that the immunodominant pattern of T cells against SARS-CoV-2 is closely associated with the expression level of viral proteins (10). To date, numerous studies have reported immunodominant T cell epitope. However, there are significant variations among these studies, including differences in screening procedures, HLA alleles considered, antigens targeted, sample sizes of individuals analyzed, and the criteria used to define “immunodominance” (12, 68–70). For example, Peng et al. reported several immunodominant peptides, defining them as those recognized by at least 6 individuals out of a pool of up to 16 subjects screened (12). Tarke et al. also highlighted the presence of highly immunodominant epitopes, with 49 HLA-II-restricted epitopes recognized by at least 3 out of an average of 10 donors, and 41 HLA-I-restricted epitopes recognized by over 50% of HLA-matched donors (68). Nielsen et al. found a broad spectrum of T cell responses, with the top three immunogenic epitopes derived from different SARS CoV-2 proteins (70). Keller et al. defined immunodominant epitopes as those recognized by multiple donors from M, N and S viral proteins (69).

**Figure 1:**
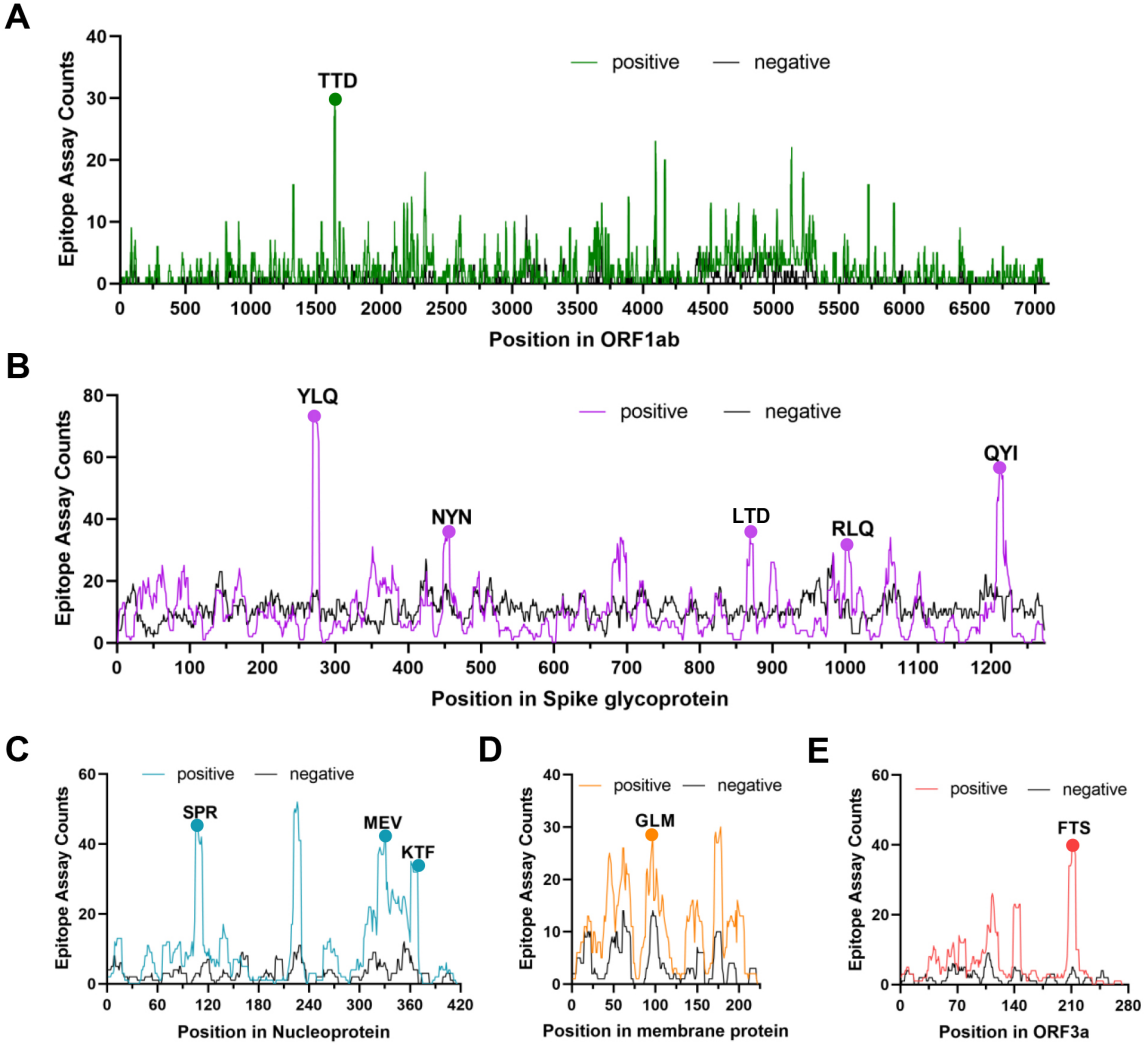
The identification of immunodominant antigenic regions of SARS-CoV-2 CTL epitopes. **(A-E)** CTL Epitope assay counts of five viral proteins (ORF1ab, Spike, N, M, and ORF3a) were analyzed using the IEDB’s Immunome Browser tool to identify potential antigenic regions. The number of positive and negative assays are indicated for each residue position.

Several independent studies, including one involving the BNT162b2 mRNA vaccine, have identified the YLQ-epitope as immunodominant (20, 23, 71–74), eliciting an immune response in the vast majority of convalescents with the HLA-A*02 genotype (responses in 16 out of 17 individuals studied) (66, 70, 71). Another notable example is the HLA-A*01:01-restricted TTD epitope (A*01/NSP3_819-828_, TTDPSFLGRY). Nelde et al. reported a positive response to the TTD epitope in 83% of donors (67). Saini et al. conducted an extensive analysis of over 3,000 peptides for 10 HLA alleles and confirmed the recognition of the same HLA-A*01:01-restricted epitope (75, 76). Additionally, dominant CD8^+^ T cell responses have been identified for the LTD (A*01/S_865-873_, LTDEMIAQY) epitope and KCY (A*03/S_378-386_, KCYGVSPTK) epitope when analyzing vaccine-elicited CD8^+^ T cell responses that span the whole S protein (77). Using the Immunome Browser tool (developed and hosted by the IEDB, https://www.iedb.org/), we plotted the epitope assay counts for each residue of SARS-CoV-2 ORF1ab, S, N, M, and ORF3a proteins. The number of positive and negative assays are indicated for each residue position, allowing for the visualization of immunodominance patterns (**Figure 1**). A range of potentially immunodominant CTL epitopes were identified on these viral proteins, some of which have been characterized by peptide-specific TCR repertoires, including ORF1ab-TTD (67, 75, 76), spike-YLQ (20, 71, 78–82), NYN (79, 83, 84), LTD (79, 84), RLQ (71, 84) (A*02/S_269-277_, RLQSLQTYV), QYI (78, 79, 82–84) (A*24/S_1208-1216_, QYIKWPWYI), nucleoprotein-SPR (80, 82, 84, 85) (B*07/N_105-113_, SPRWYFYYL), MEV (82) (B*40/N_322-331_, MEVTPSGTWL), KTF (82, 86) (A*03/A*11/N_361-369_, KTFPPTEPK), and ORF3a-FTS (79, 84) (A*01/ORF3a_207-215_, FTSDYYQLY) (**Table 2**). Meanwhile, we also observed that some potentially dominant epitopes, such as membrane protein-GLM (A*02/M_89-97_, GLMWLSYFI) and ORF3a-FTS, have emerged several mutations associated with CTL immune escape, including GLM-L90F and FTS-Q213K mutations (20, 88) (**Table 3**). These dominant epitopes warrant further investigation, as they may offer valuable insights for developing effective SARS-CoV-2 vaccines.

**Table 2:** TCR epitope characteristics of 22 SARS-CoV-2 epitopes in the public VDJ database (87).

Name	Epitope	Location	HLA-I allele	Major TRAV (%), TRAJ (%) TRBV (%), TRBJ (%)	Count (TRAV, AJ, BV, BJ)	Reference
YLQ	YLQPRTFLL	S_269-277_	A*02	**AV12-1 (53%)**, AJ43 (23%), BV7-9 (18%), BJ2-2 **(57%)**	872, 864, 1110, 1110	(20, 71, 78–82)
SPR	SPRWYFYYL	N_105-113_	B*07:02	AV4 (11%), AJ10 (8%), BV27 (18%), BJ2-7 (20%)	467, 449, 608, 608	(80, 82, 84, 85)
TTD	TTDPSFLGRY	NSP3_819-828_	A*01:01	AV9-2 (12%), AJ49 (8%), BV27 (28%), BJ2-7 (21%)	407, 394, 437, 437	(79, 82)
KTF	KTFPPTEPK	N_361-369_	A*03:01, A*11:01	AV19 (9%), AJ12/4249 (5%), BV20-1 (9%), BJ2-2 (17%)	135, 132, 179, 179	(82, 86)
QYI	QYIKWPWYI	S_1208-1216_	A*24:01, A*24:02	AV19 (13%), AJ25 (7%), BV20-1 (25%), BJ2-7 (30%)	142, 136, 158, 158	(78, 79, 82–84)
LTD	LTDEMIAQY	S_865-873_	A*01:01	AV21 (14%), AJ40 (10%), BV27 (18%), BJ2-7 (21%)	135, 125, 135, 135	(79, 84)
MEV	MEVTPSGTWL	N_322-331_	B*40:01	AV14 (35%), AJ4 (25%), BV27 (31%), BJ2-1 (29%)	74, 73, 90, 90	(82)
NQK	NQKLIANQF	S_919-927_	B*15:01	AV21 (14%), AJ40 (18%), BV7-2 (10%), BJ1-2 (25%)	77, 72, 77, 77	(79)
RLQ	RLQSLQTYV	S_1000-1008_	A*02	AV16/38-1/28-2 (11%), A20 (19%) BV27 (31%), BJ2-1 (29%)	54, 54, 94, 94	(71, 84)
NYN	NYNYLYRLF	S_448-456_	A*24:01, A*24:02	**AV12-1 (75%)**, AJ28 (19%) BV6-1 (27%), **BJ2-7 (80%)**	57, 57, 70, 70	(79, 83, 84)
LLY	LLYDANYFL	ORF3_139-147_	A*02:01	**AV8-1 (65%)**, **AJ29 (56%)** BV11-2 (38%), **BJ1-1 (56%)**	34, 34, 55, 55	(79, 84)
FTS	FTSDYYQLY	ORF3_207-215_	A*01:01	AV14 (21%), AJ52 (13%) BV7-3 (14%), BJ2-7 (23%)	42, 39, 43, 43	(79, 84)
PTD	PTDNYITTY	NSP3_1321-1329_	A*01:01	**AV12-1 (46%)**, **AJ24 (49%)** **BV28 (51%)**, **BJ2-7 (61%)**	37, 37, 41, 41	(79, 84)
ALS	ALSKGVHFV	ORF3_72-80_	A*02:01	AV1-2 (13%), AJ24 (23%) BV27 (21%), BJ2-1 (30%)	24, 22, 43, 43	(79, 84)
ALW	ALWEIQQVV	ORF1ab_4094-4102_	A*02:01	AV19 (19%), AJ42 (19%) BV19 (14%), BJ2-1 (22%)	27, 26, 36, 36	(84)
VYF	VYFLQSINF	NSP3_112-120_	A*24:02	*, AJ30 (18%) BV19 (17%), BJ2-7 (21%)	29, 28, 29, 29	(79, 84)
RVA	RVAGDSGFAAY	M_186-196_	B*15:01	AV1-2 (21%), AJ43/45 (14%) BV27/7-9 (17%), BJ1-5/2-1/2-7 (17%)	24, 22, 24, 24	(79)
KLW	KLWAQCVQL	ORF1ab_3886-3894_	A*02:01	**AV38-2 (76%)**, AJ43 (29%) BV6-6 (20%), BJ2-1 (36%)	21, 21, 25, 25	(84)
KSV	KSVNITFEL	ORF1ab_837-845_	A*02:01	AV5 (19%), AJ39/49 (13%) *, BJ2-1 (26%)	16, 16, 19, 19	(84)
WPV	WPVTLACFV	M_58-66_	B*07:02	AV38-1 (14%), * BV7-9 (14%), BJ2-7 (19%)	14, 14, 21, 21	(84)
SII	SIIAYTMSL	S_691-699_	B*07:02	AV14 (19%), AJ54/9 (13%) BV4-2 (11%), BJ2-3 (33%)	16, 15, 18, 18	(84)
VWV	VMVELVAEL	ORF1ab_84-92_	A*02:01	AV13-1/13-2 (15%), AJ5 (17%) BV27 (18%), BJ2-1/2-7 (24%)	13, 12, 17, 17	(84)

*, not available. Bold values indicate TCR genes that constitute more than 50% of the peptide-specific TCR repertoire.

**Table 3:** CTL immune escape by SARS-CoV-2 mutations.

Original sequence	Location	HLA	Mutation	Mutant sequence	CTL immune escape	Reference
YLQPRTFLL	S_269-277_	A*02	L270F P272L	Y **F** QPRTFLL YLQ **L** RTFLL	IFN-γ^+^ CD8^+^ T cells **↓** peptide-MHC-I binding **↓** tetramer^+^ CD8^+^ T cells **↓** specific-TCR binding **↓** TNF^+^ CD8+ T cells **↓** MIP-1β release **↓** CD107a release **↓** T cell activation **↓**	(20, 28, 29)
RLQSLQTYV	S_1000-1008_	A*02	T1006I	RLQSLQ **I** YV	specific-TCR binding **↓** T cell activation **↓**	(28)
FVFLVLLPLV	S_2-11_	A*02	L5F L8V	FVF **F** VLLPLV FVFLVL **V** PLV FVF **F** VL **V** PLV	peptide-MHC-I binding **↓** tetramer^+^ CD8^+^ T cells **↓** CD69^+^ CD8^+^ T cells **↓** CD137^+^ CD8^+^ T cells **↓**	(89)
FQFCNDPFL	S_133-141_	A*02	D138H/Y	FQFCN **H** PFL FQFCN **Y** PFL	peptide-MHC-I binding **↓**	(89)
YQDVNCTEV	S_612-620_	A*02	D614G	YQ **G** VNCTEV	peptide-MHC-I binding **↓**	(89)
FTSDYYQLY	ORF3a_207-215_	A*01:01	Q213K	FTSDYY **K** LY	IFN-γ ELISpot **↓** killing capacity **↓**	(88)
QRNAPRITF	N_9-17_	B*27:05	P13L/S/T	QRNA **L** RITF QRNA **S** RITF QRNA **T** RITF	IFN-γ ELISpot **↓** killing capacity **↓**	(88)
KTFPPTEPK	N_361-369_	A*03:01 A*11:01	T362I P365S	K **I** FPPTEPK KTFP **S** TEPK	IFN-γ ELISpot **↓** killing capacity **↓**	(88)
TTDPSFLGRY	ORF1a_1637-1646_	A*01:01	T1637I T1638I P1640S P1640L P1640H	**I** TDPSFLGRY T **I** DPSFLGRY TTD **S** SFLGRY TTD **L** SFLGRY TTD **H** SFLGRY	IFN-γ^+^ CD8^+^ T cells **↓** CD107a^+^ CD8^+^ T cells **↓** TNFα^+^ CD8^+^ T cells **↓** killing capacity **↓**	(88)
YFPLQSYGF	S_489-497_	A*29:02	Q493R G496S	YFPL **R** SY **S** F	IFN-γ^+^ CD8^+^ T cells **↓** IFN-γ^+^ TNF^+^ CD8^+^ T cells **↓**	(30)
PTDNYITTY	OEF1ab_1321-1329_	A*01:01	T1322A T1322P	P **A** DNYITTY P **P** DNYITTY	IFN-γ^+^ CD8^+^ T cells **↓**	(90)
NYNYLYRLF	S_448-456_	A*24:01 A*24:02	L452R Y453F	NYNY **R** YRLF NYNYL **F** RLF	IFN-γ^+^ CD8^+^ T cells **↓**	(91, 92)
KIADYNYKL	S_417-425_	A*02:01	K417N	**N** IADYNYKL	IFN-γ^+^ CD8^+^ T cells **↓**	(92)
GVYYHKNNK	S_142-150_	A*11:01	Y144del	GVYHKNNK	IFN-γ^+^ CD8^+^ T cells **↓**	(92)
IIWFLLLSV	ORF1ab_2230-2238_	A*02:01 A*02:07	I2230T	**T** IWFLLLSV	IFN-γ^+^ CD8^+^ T cells **↓**	(92)
GLMWLSYFI	M_89-97_	A*02:01	L90F	G **F** MWLSYFI	peptide-MHC-I binding **↓** tetramer^+^ CD8^+^ T cells **↓** IFN-γ^+^ CD8^+^ T cells **↓**	(20)
MEVTPSGTWL	N_322-331_	B*40:01	M322I L331F L331S T325I	**I** EVTPSGTWL MEVTPSGTW **F** MEVTPSGTW **S** MEV **I** PSGTWL	peptide-MHC-I binding **↓**, tetramer^+^ CD8^+^ T cells **↓** IFN-γ^+^ CD8^+^ T cells **↓**	(20)
LLFNKVTLA	S_821-829_	A*02	L822F	L **F** FNKVTLA	peptide-MHC-I binding **↓**	(20, 88)
SIIAYTMSL	S_691-699_	B*07:02	S691P/C I692T	**P** IIAYTMSL **C** IIAYTMSL S **T** IAYTMSL	peptide-MHC-I binding **↓**	(20)

IFN-γ, interferon-gamma; TNF, tumor necrosis factor; CD107a, cluster of differentiation 107a; MIP-1β, macrophage inflammatory protein-1 beta. The bold, colored, and underlined letters in the mutant sequences indicate mutation sites on CTL epitopes.

The downward arrow indicates a reduction in the population of activated CD8+ T cells, a decrease in cytokine secretion, or a weakening of peptide-MHC/peptide-TCR binding.

## 4 Biological insights into COVID-19 from the TCR repertoire

**Figure 2:**
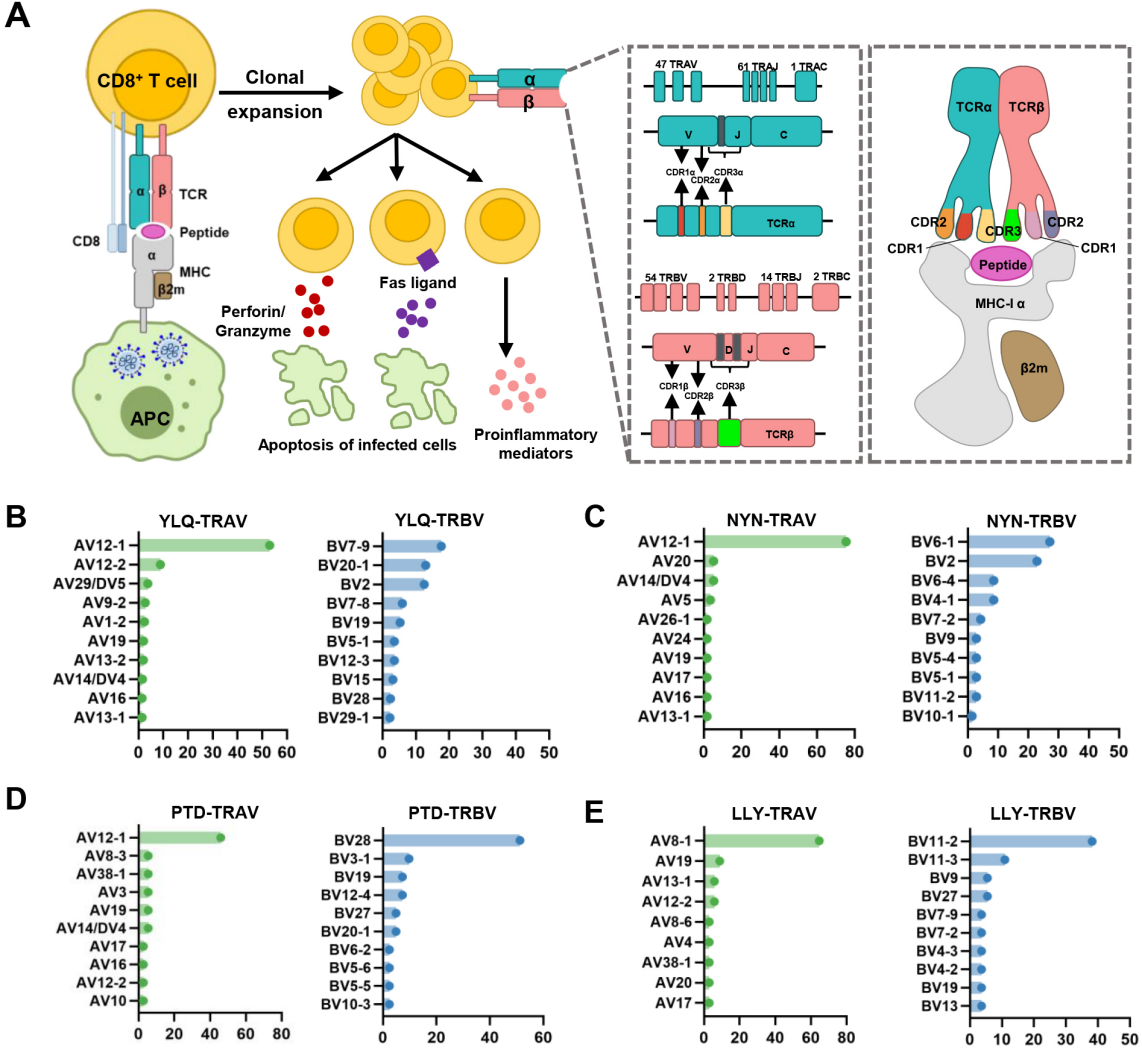
Overview of CTL response to SARS-CoV-2 infection mediated by TCR-pMHC complex. **(A)** Antigen presenting cells (APCs) endocytose SARS-CoV-2 and degrade it through antigen processing. These epitope fragments are then presented on the cell surface by MHC molecules and allow recognition by T cells. TCR genes of the α-chain (TCRα) and β-chain (TCRβ) on the T cell surface are recombined to produce a diverse TCR repertoire. If a CD8^+^ T cell is able to bind pMHC, it will undergo clonal expansion and directly target infected cells through perforin/granase, FAS ligand/tumor necrosis factor-related apoptosis-inducing ligand (TRAIL) pathway, or secretion of pro-inflammatory mediators. CDR1 and CDR2 are encoded by TRBAV and TRABV genes, and CDR3 encompasses VJ regions (for TCRα) or VDJ regions (for TCRβ). Non-template nucleotide insertions and deletions are represented by black boxes. MHC-α (green), β2m (sand), Peptide (magenta), TCRα (teal), TCRβ (salmon), TCRα-CDR1 (red), TCRα-CDR2 (orange), TCRα-CDR3 (yellow), TCRβ-CDR1 (blue), TCRβ-CDR2 (green), TCRβ-CDR3 (maroon). **(B–E)** Histograms of V gene usage across the sequences of YLQ-, NYN-, PTD-, LLY-specific TCRs. Only the top ten of each gene are shown.

The specificity of T cells toward viral antigens presented by MHCs is determined by unique TCRs (93). TCRs exhibit considerable sequence heterogeneity due to somatic recombination of different variable (V), diversity (D), and joining (J) gene fragments, as well as random mutations of nucleotides at segment junctions (94). As a result, each TCR chain possesses three variable complementary determination regions (CDRs): the germline-encoded CDR1 and CDR2 loops, and the hypervariable CDR3 loop (**Figure 2A**). Upon antigen recognition, activated T cells undergo rapid clonal expansion, resulting in a substantial increase in T cells with identical TCRs, thus producing the identical antigen recognition (95). Expanded CTL clones possess specific TCRs for viral antigens, directly lyse infected cells via perforin/granzyme release, and secrete pro-inflammatory mediators (87). Hence, investigating the role of TCR in SARS-CoV-2 infection should garner vast interest.

### 4.1 Interindividual TCR repertoire influencing immune responses

It is well well-established that TCR diversity declines with age. In individuals within the first two decades of life, TCRβ diversity in the naïve T cell repertoires is estimated to be 60-120 million, but a decline to 8-57 million is observed in individuals over 70 years of age (96, 97). This age-related decline in TCR repertoire has been confirmed in the antiviral responses to human influenza A viruses (98, 99). This age-related decline in TCR repertoire has been observed in the antiviral responses to human influenza A viruses (98, 99). The numbers of antigen-specific CD8^+^ T cells across universal influenza epitopes were reduced in the elderly, although their effect/memory phenotype remained stable (98). Interestingly, the mortality rate of elderly COVID-19 patients is notably higher than that of young and middle-aged patients, while children mostly exhibit milder disease outcomes when infected with SARS-CoV-2 (2, 100, 101). Further studies are needed to clarify whether aged and less diverse TCR repertoires impact the ability of elderly patients to generate sufficiently robust T cell response to SARS-CoV-2. HLA variation is another factor of concern, influencing the composition of TCR genes by affecting both intra-thymus and extra-thymus clonal selection (102). A study by Francis et al. demonstrated that HLA variations significantly affect the CD8^+^ T cell repertoire shape and utilization of immune recall upon SARS-CoV-2 infection (84). Genetic differences in the HLA genes directly affect the binding affinity of MHC molecules to antigens, which in turn confer susceptibility or resistance to viral infections (32, 33, 103).

### 4.2 Diversity of TCR repertoire after SARS-CoV-2 infection and vaccination

The size, frequency, and publicity of individual clonotypes within the TCR repertoire can provide insights into both successful and failed immune responses. During SARS-CoV-2 infection, the diversity and clonability of TCR repertoire peaked within 8-14 days (104, 105), and then returned to base levels within a week after virus elimination (106). In COVID-19 convalescent individuals, SARS-CoV-2 peptide continue to mediate long-term immune responses, with robust functional T cell responses persisting for up to 6 months post-infection (107). A study by Cohen et al. evaluated 254 COVID-19 patients longitudinally up to 8 months and found that virus-specific CD4^+^ and CD8^+^ T cells were polyfunctional and maintained with an estimated half-life of 200 days (108). Long-term immunity against SARS-CoV-2 is primarily driven by the clonal diversity of antigen-specific T cell responses (107, 109). Studies have demonstrated that highly diverse TCR repertoires can offer protection against a variety of antigens, including those from CMV, EBV, and HIV (110, 111), and such repertoires may be associated with a higher level of affinity, affinity, and overall functionality in immune responses (112, 113).

Vaccination against SARS-CoV-2 infection elicit strong T cell responses. Vaccine-induced CTL expansion seems to be relatively weak and results in fewer distinct clonotype clusters compared to CTLs induced by natural infection (114). Despite the rapid contraction of the circulating T Cell responses to SARS-CoV-2 mRNA vaccination, there is a persistent memory that were readily detectable in most individuals out to 235 days after vaccination (115). Repeated mRNA vaccination lead to large expansions of memory spike-reactive T cell clonotypes, most of which were CD8^+^ T cells, while also eliciting diverse spike-reactive T cell clonotypes not observed before vaccination (116). Infection-induced spike-specific CD8^+^ T cell memory plays an important role in the formation of circulating T cell bank size and clonal composition after vaccination (117), and mRNA vaccination promotes the expansion of memory CD8^+^ T cells (117, 118). As both virus- and vaccine-induced antigen-specific TCR repertoires undergo significant clonal contraction over time, coupled with an overall decline in immune response, booster vaccination may be the primary strategy to enhance long-term protection (109, 119).

Viral infection triggers massive T cells that can recognize specific antigens, resulting in skewing of the TCR repertoire toward these antigen-specific T cells (120). It has been demonstrated that certain V, D, and J fragments are over-expressed or under-expressed in COVID-19 patients with different clinical pictures (121). Compared with symptomatic patients, asymptomatic patients exhibit an overrepresentation of some TCR genes, including TRAV (AV17, AV12-1, AV19, AV35, and AV41), TRBV (BV12-5 and BV19), TRAJ16, and TRBJ2-1 (122, 123). Symptomatic patients, on the other hand, have higher frequencies of TRAV2, AJ8, AJ40, BV3-1, and BV5-1 (122). Severe patients tend to highly express TRBV5-6, BV14, BV13 and BV24-1 (124). Furthermore, a study showed that 25 sequences within the central parts of CDR3 region could be used to predict severe infection, emphasizing the significant impact of distinct clonal expansion on disease progression (125).

### 4.3 SARS-CoV-2 CTL epitopes recognized by public and private TCRs

Interestingly, despite an estimated potential TCR diversity of 10^15^ (126, 127), TCRs that recognize a common ligand typically exhibit convergent sequence features in CDR3 residues that directly contact the peptide (111, 128), as well as CDR1 and CDR2 residues that can also contact the peptide and MHC, which are also known as “public” TCR motifs (129). In previous HIV-related studies, viral control has been linked to the presence of more cross-reactive public TCR clones, which may play a role in limiting viral escape pathways (130, 131). Several studies have identified “public” TCR in COVID-19 convalescents, characterized by conserved CDR motifs within and between individuals (71, 104, 132, 133). Ford et al. identified public CD8^+^ and CD4^+^ TCR motifs associated with SARS-CoV-2 spike specificity through TCR sequence similarity clustering (116). Analysis of over 4,000 epitope-specific TCR sequences showed that all SARS-CoV-2 exposures elicit diverse repertoires characterized by shared TCR motifs, confirmed by monoclonal TCR characterization (134). Here, we summarized TCR repertoires for 22 epitopes (each with a gene count >30) from the public VDJ database (135), as shown in **Table 2**. Consistent with previous studies (71, 133, 136), we found that TRAV12-1 (53%, 462/872) and TRBV7-9 (18%, 196/1110) were used by most YLQ-specific TCRs (**Figure 2B**). Likewise, TCRs recognizing other SARS-CoV-2 epitopes (e.g., NYN, LLY, PTD) display notable bias in the usage of specific TCR gene fragments (**Figures 2C–E**). For example, TRAV12-1 gene was commonly employed by YLQ, NYN, and PTD specific TCRs. Half of the epitopes we counted, such as SPR, TTD, QYI, LTD, RLQ, NYN, FTS, PTD, VYF, RVA, and WPV were dominated by the TRBJ2-7 gene (**Table 2**). This strong bias of TCR gene usage among epitope-dependent TCRs likely highlights the significance of germline-encoded features in TCR recognition, as previously reported in other antiviral immune responses (137, 138).

In addition to public TCRs, the TCR repertoire generated in response to a specific epitope varies between individuals, often referred to as “private” responses. The RLQ-epitope (spike_1000-1008_, RLQSLQTYV) is another immunodominant epitopes located on the SARS-CoV-2 spike protein, triggering cellular responses in most HLA-A*02:01^+^ convalescents (71). Unlike the highly “public” TCRs generated in response to the YLQ-epitope (71), TCRs responding to RLQ epitopes tend to be predominantly “private” and exhibit greater diversity (**Table 2**). The recognition of A*02-RLQ by receptors with diverse CDR3α and CDR3β pairings diminishes the publicity of A*02-RLQ responses between individuals, enabling them to recognize MHC and peptide in a manner that reduces the likelihood of identical or very similar V(D)J rearrangements in different individuals (28).

### 4.4 Cross-reactiveness of T cell repertoire in human coronavirus

Based on the genomic similarities between SARS-CoV-2 and HCoVs, cross-reactive T cells may underlie the extensive heterogeneity observed in COVID-19 disease. SARS-CoV-2 T cell reactivity was mostly associated with CD4^+^ T cells, with a smaller contribution by CD8^+^ T cells (9, 10, 139, 140). Nevertheless, some reports provide the basis for a limited representation of cross-reactive CD8^+^ T cell responses (19, 60, 84, 85, 141). Minervina et al. detected certain T cell clones in memory fragments at pre-infection time points, suggesting participation of pre-existing cross-reactive memory T cells in the immune response to SARS-CoV-2 (19). Francis et al. reported that the clonal diversity of T cell responses to HLA-B*07:02 allele correlates with pre-existing immunity, allowing efficient presentation of homologous epitopes from both SARS-CoV-2 and HCoVs (84). Cytotoxic T cell responses against SPR-HLA-B*07:02 (N_105-113_, SPRWYFYYL) are often associated with mild cases of COVID-19 (80, 84, 142–145). Augusto et al., found that T cells from pre-pandemic samples from individuals carrying HLA-B*15:01 were reactive to the immunodominant SARS-CoV-2 spike-derived NQK epitope (60). Additionally, Shimizu et al. found that CD8^+^ T cells in response to a selected dominant QYI epitope display multifunctionality and cross-functionality across HCoVs in HLA-A24^+^ donors (141).

**Figure 3:**
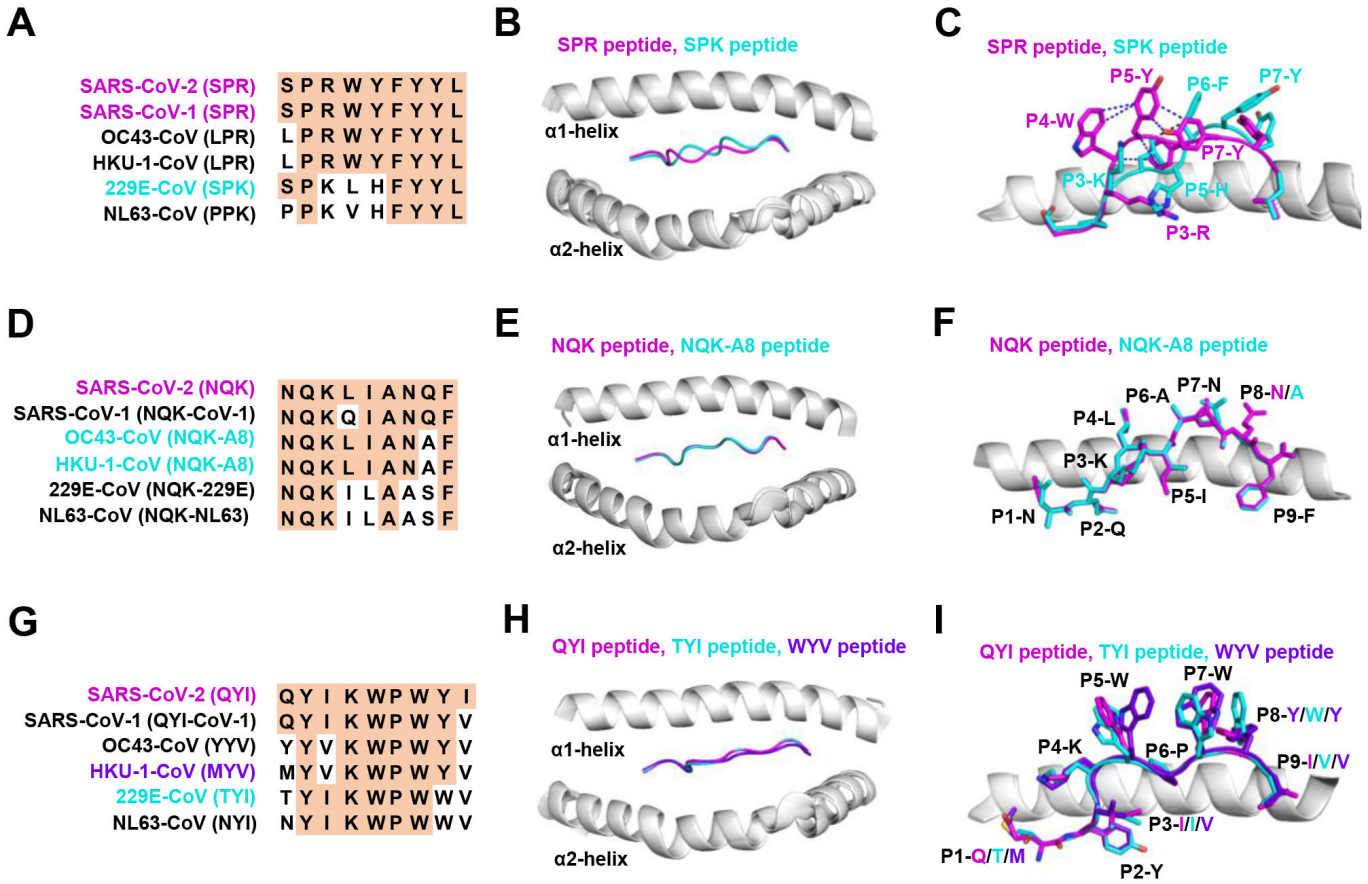
The structural basis of SPR, NQK, and QYI peptides for selective T cell cross-reactivity. **(A)** Peptide homologs of other HCoVs compared to the SARS-CoV-2 SPR peptide. **(B)** Structural superposition of the SPR-HLA-B7 (ID 7LGD) and SPK-HLA-B7 (ID 7LGT). SPR peptide (magenta), SPK peptide (cyan), HLA-B7 (grey). **(C)** Top view of the SPR-HLA-B7 and SPK-HLA-B7, with stick representation of the SPR and SPK peptides. Blue and black dashed lines indicate intra-peptide interactions of the SPR and SPK peptide, respectively. **(D)** Peptide homologs of other HCoVs compared to the SARS-CoV-2 NQK peptide. **(E)** Structural superposition of the NQK-HLA-B15 (ID 8ELH) and NQK-A8-HLA-B15 (ID 8ELG). NQK peptide (magenta), NQK-A8 peptide (cyan), HLA-B15 (grey). **(F)** Top view of the SPR-HLA-B7 and SPK-HLA-B7, with stick representation of the SPR and SPK peptides. **(G)** Peptide homologs of other HCoVs compared to the SARS-CoV-2 QYI peptide. **(H)** Structural superposition of the QYI-HLA-A24 (ID 7EJL), TYI-HLA-A24 (ID 7EJM), and MYV-HLA-A24 (ID 7EJN). QYI peptide (magenta), TYI peptide (cyan), MYV peptide (purple blue), HLA-A24 (grey). **(I)** Top view of the QYI-HLA-A24, TYI-HLA-A24, and MYV-HLA-A24, with stick representations of the QYI, TYI and MYV peptides.

The HLA-B*07:02-restricted SPR epitope is one of the most cross-reactive epitopes, showing a high frequency in unexposed pre-pandemic samples (80, 145). Notably, the SPR peptide sequence is identical in SARS-CoV-2 and SARS-CoV-1, differing by only one residue in OC43-CoV-1 and HKU-1-CoV-1 (LPR), three residues in 229E (SPK) and four in NL63-CoV-1 virus (PPK), as shown in **Figure 3A**. Lineburg et al. reported that CD8^+^ T cells exhibit cross-reactivity between SARS-CoV-2 SPR and OC43/HKU-1-derived LPR peptide, but CD8^+^ T cells were unable to cross-recognize SPK and PPK peptides (85). The crystal structures of SPR-HLA-B*07:02 and SPK-HLA-B*07:02 elucidate these outcomes. Despite sharing common motifs in P1-2 (SP) and P6-9 (FYYL), differences in peptide sequences at P3-5 result in distinct conformations for SPK and SPR (**Figure 3B**). In the structure of SPR-HLA-B*07:02, four of the five aromatic residues create an interaction network, forming a compact and substantial binding surface for potential interaction with TCRs (**Figure 3C**). In comparison, the SPK peptide predominantly exposes the three carboxyl residues (P6-8) at the C-terminus (**Figure 3C**). Due to their high sequence identity, the LPR peptide (from OC43 and HKU-1) may adopt a conformation similar to that of SPR peptide (found in SARS-CoV-1 and SARS-CoV-2), providing the foundation for cross-reactivity among CD8^+^ T cells in HLA-B7^+^ individuals. However, despite sharing >55% (5/9) sequence identity, the distinctive structures account for the relatively low-level CD8^+^ T cell cross-reactivity observed between these peptides.

Most NQK-specific reactive T cells display a memory phenotype, exhibit highly polyfunctional, and cross-react to a peptide from seasonal coronaviruses (60, 61). The NQK peptide of SARS-CoV-2 differs by only one residue in SARS-CoV-1, OC43-CoV-1, and HKU-1-CoV-1, and by three residues in 229E-CoV-1 and NL63-CoV-1 virus (**Figure 3D**). Crystal structures of peptide-HLA-B15 complexes reveal that the peptides NQKLIANQF (from SARS-CoV-1) and NQKLIANAF (NQK-A8, from OC43-CoV and HKU1-CoV) have similar stabilization properties (**Figures 3E, F**). This structural similarity of the peptides underpins T cell cross-reactivity of high-affinity public T cell receptors, providing a molecular basis for HLA-B*15:01-mediated pre-existing immunity (60). A similar pattern of peptide binding to HLA was observed with another dominant epitope, HLA-A24 restricted QYI (141). The QYI exhibits high sequence homology with SARS-CoV-1, OC43 (YYV), HKU-1-CoV-1 (MYV), 229E-CoV-1 (TYI), and NL63-CoV-1 (NYI) (**Figure 3G**). The cross-reactivity of the QYI epitope depends on the structural pattern of the peptide-HLA-A*24:02 complex and the combinations of TCR sequences (141). The structures of QYI-HLA-A24, TYI-HLA-A24, and MYV-HLA-A24 are almost identical, with rmsd (root mean square deviation) of 0.15 Å (QYI and TYI), 0.41 Å (QYI and MYV), and 0.41 Å (TYI and MYV), respectively (**Figure 3H**). The side chains of the peptides at P1, P4, P5, P7 and P8 are directed to the solvent region, and their structural orientations are slightly different (**Figure 3I**). This particular region forms a raised structure and is expected to interact directly with TCRs by their side chains. It is reasonable to speculate that some CD8^+^ T cells targeting seasonal coronaviruses may exist as long-term memory cells within the population. If these cross-reactive T cells are stimulated by COVID-19 vaccine or viral antigen, they could be skewed toward SARS-CoV-2.

## 5 CD8 ^+^ T cell immune escape by SARS-CoV-2 variants

### 5.1 Antigenic mutations and loss of CTL epitope-specific responses

Given the active role of T cells in combating viral infection, some mutations could potentially lead to the loss of CTL epitopes that evade recognition by CD8^+^ T cells (**Table 3**). Mutations within CD8^+^ epitopes in S protein (YLQ-L270F, LLF-L822F, and SII-S691P/S691C/I692T), M protein (GLM-L90F), N protein (MEV-M332I/L331S/L331F) were noted in one study during the course of acute infections, resulting in loss of epitope-specific responses (20). Prolonged SARS-CoV-2 infection in immunocompromised hosts may create a great opportunity for T cell escape. For instance, in the case of chronic SARS-CoV-2 infection, the NSP3 T504P mutation has been reported to result in the loss of CTL response (90, 146). These findings are limited to a few cases and suggest the need for more prospective cohort studies to systematically assess the risk of T cell escape in certain patient populations.

As the T cell responses target epitopes across the SARS-CoV-2 genome, the footprint of T cell escape is more broadly distributed than antibody-driven changes. In multiple SARS-CoV-2 lineages, some mutations within the immunodominant ORF1a (TTD- T1637I, T1638I, P1640S, P1640L, P1640H) ORF3a (FTS-Q213K) and N protein (QRN-P13L/S/T, KTF-T362I, P365S) CD8^+^ T cell epitopes resulted in a complete loss of recognition (88). Among these mutations, the N protein P13L mutation is present in Omicron within B*27:05-restricted CD8^+^ epitopes. Given the hypothesis that VOCs arise in chronic infections, it is tempting to speculate that the presence of P13L in Omicron reflects selection due to T cell stress during chronic infection, in addition to the constellation of spike mutations that are likely driven by antibody pressure. The mutant YLQ-P272L epitope could not be recognized by over 120 YLQ-specific TCRs, which may allow viral variants to escape from vaccine-induced T cell responses (28, 29). Spike-encoded L452R and Y453F led to the loss of HLA-A24-restricted CTL responses (91). In addition to evading antibodies and enhancing ACE2 binding affinity (147), the role of T cells in driving this change is uncertain. The extent to which these observations also represent incidental effects of mutations driven by other stresses on T cell responses is currently unknown.

### 5.2 Mechanisms of CTL immune escape by SARS-CoV-2 variants

**Figure 4:**
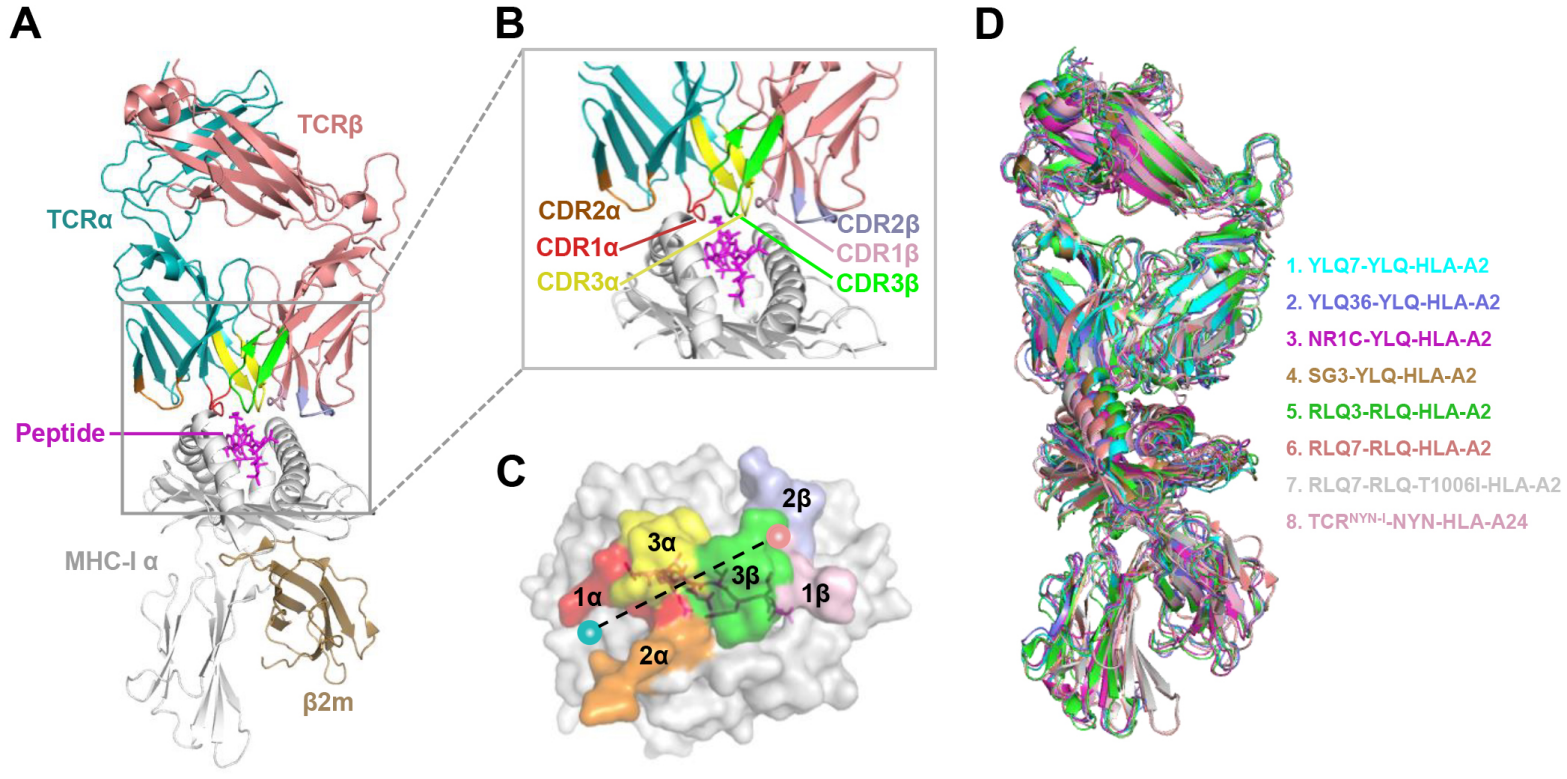
Schematic view of the TCR-pMHC I ternary complex. **(A, B)** Side view of YLQ7-YLQ-HLA-A2 (ID 7N1F). MHC-α (grey), β2m (sand), YLQPRTFLL-peptide (magenta), TCRα (teal), TCRβ (salmon), CDR1α (red), CDR2α (orange), CDR3α (yellow), CDR1β (light pink), CDR2β (light blue), CDR3β (green). **(C)** Footprint of TCR YLQ7 on YLQ-HLA-A2. **(D)** Cartoon comparison of eight TCR-pMHC ternary complexes presenting SARS-CoV-2 CD8^+^ T cell epitopes, including YLQ7-YLQ-HLA-A2 (ID 7N1F, cyan), YLQ36-YLQ-HLA-A2 (ID 7PBE, slate), NR1C-YLQ-HLA-A2 (ID 7N6E, magenta), SG3-YLQ-HLA-A2 (ID 7RTR, sand), RLQ3-RLQ-HLA-A2 (ID 7N1E, green), RLQ7-RLQ-HLA-A2 (ID 8GOM, salmon), RLQ7-RLQ-T1006I-HLA-A2 (ID 8GON, grey), TCR^NYN-I^-NYN-HLA-A24 (ID 8YE4, light pink).

T cell escape can occur through several mechanisms. Amino acid changes within epitopes or flanking regions can disrupt antigen processing (148), and changes to anchor residues can interfere with MHC/TCR binding to epitopes (29, 92, 149). Both these mechanisms can result in irreversible loss of T cell responsiveness to a particular epitope. To better understand the mechanism of CTL immune escape achieved by alterations in the binding between ligands and receptors, researchers have resolved several crystal structures of TCR-pHLA ternary complex and pHLAs loaded with original or mutant SARS-CoV-2 peptides, including YLQ, YLQ-P272L, RLQ, RLQ-T1006I, NYN, NYN-Y453F, KIA, KIA-K417T peptides (**Table 4**). All of eight TCR-pMHC ternary complexes loaded with SARS-CoV-2 peptides are symmetrically docked on pHLAs in a canonical diagonal orientation (**Figure 4**).

**Table 4:** Published crystal structures of TCR-pHLA and pHLA associated with SARS-CoV-2 CTL immune escape.

Peptide sequence	pHLA (ID)	TCR (ID)	TCR-pHLA complex (ID)	TRAV, TRAJ, TRBV, TRBJ
YLQPRTFLL	YLQ-HLA-A2 (7P3D, 7N1A, 7RDT, 7N6D)	YLQ7 (7N1D)	YLQ7-YLQ-HLA-A2 (7N1F)	AV12-2, AJ30, BV7-9, BJ2-7
		*	NR1C-YLQ-HLA-A2 (7N6E)	AV12-1, AJ43, BV19, BJ2-2
		*	YLQ36-YLQ-HLA-A2 (7PBE)	AV12-1, AJ34, BV7-9, BJ2-2
		*	SG3-YLQ-HLA-A2 (7RTR)	AV12-2, *, BV7-9, *
YLQ **L** RTFLL	YLQ-P272L-HLA-A2 (7P3E)	*	*	*
RLQSLQTYV	RLQ-HLA-A2 (7N1B)	RLQ3 (7N1C)	RLQ3-RLQ-HLA-A2 (7N1E)	AV16, AJ39, BV11-2, BJ2-3
		RLQ7 (8GOP)	RLQ7-RLQ-HLA-A2 (8GOM)	AV38-2, AJ29, BV12-3, BJ2-3
RLQSLQ **I** YV	*	RLQ7 (8GOP)	RLQ7-RLQ-T1006I-HLA-A2 (8GON)	AV38-2, AJ29, BV12-3, BJ2-3
NYNYLYRLF	NYN-HLA-A24 (7F4W)	*	TCR^NYN-I^-NYN-HLA-A24 (8YE4)	AV12-1, AJ28, BV6-1, BJ2-7
NYNYL **F** RLF	NYN-Y453F-HLA-A24 (8ZV9)	*	*	*
KIADYNYKL	KIA-HLA-A2 (7EU2)	*	*	*
**T** IADYNYKL	TIA-HLA-A2 (7UM2)^a^	*	*	*

*, Not available. ^a^, to be published.

Reference: (7N1A, 7N1B, 7N1C, 7N1D, 7N1E, 7N1F) (28), (7P3D, 7P3E, 7PBE) (29), (7N6D, 7N6E) (150), (7RTD, 7RTR) (81), (8GOM, 8GON, 8GOP) (151), (7F4W, 7UM2) (92), (8ZV9, 8YE4) (149). The bold, colored, and underlined letters in the mutant sequences indicate mutation sites on CTL epitopes.

Mutations within the epitope may destabilize or reassemble the pHLA complexes (20, 92). For example, our previous study showed that the K417N and Y144^del^ mutations lead to failure in the formation of the KIA-HLA-A2 and GVY-HLA-A11 complexes, respectively, blocking the first step in antigen presentation (92). Additionally, structural analysis shows that the positively charged side chain of N-terminal lysine forms a π-cation interaction with the indole ring of W167 of HLA-A2, and K417N/T mutations at this position (K417T) are expected to abolish the π-cation interaction at this position (92). The loss of this key peptide-HLA interactions may provide SARS-CoV-2 variants with an opportunity to evade cellular immunity.

**Figure 5:**
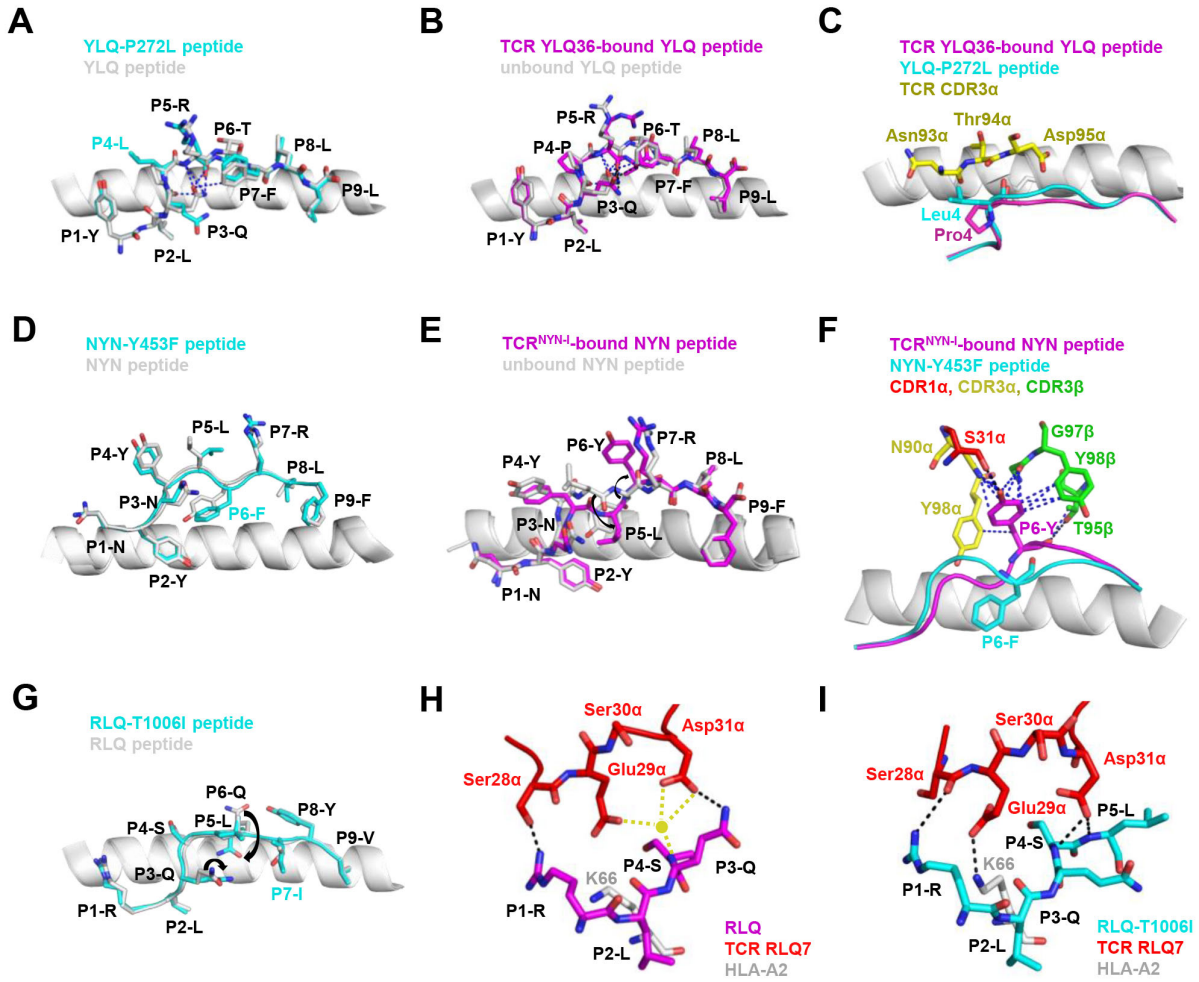
Mechanism of CTL immune evasion mediated by SARS-CoV-2 epitope mutation. **(A)** Comparison of YLQ-HLA-A2 (ID 7P3D) and YLQ-P272L-HLA-A2 (ID 7P3E). Intrapeptide bonds present in YLQ-HLA-A2 are shown as blue dashes. YLQ peptide (grey sticks), YLQ-P272L peptide (cyan sticks), HLA-A2 (grey cartoon). **(B)** Comparison of unbound YLQ-HLA-A2 (ID 7P3D) and TCR-bound YLQ-HLA-A2 (ID 7PBE) peptide presentation. Intrapeptide bonds present in TCR-unbound and TCR-bound YLQ-HLA-A2 are shown as blue and black dashes, respectively. TCR-bound YLQ peptide (magenta), TCR-unbound YLQ peptide (grey sticks), HLA-A2 (grey cartoon). **(C)** YLQ and YLQ-P272L P4 residues shown as magenta and grey sticks, respectively. YLQ36 CDR3α loop shown as yellow sticks. **(D)** Comparison of NYN-HLA-A24 (ID 7F4W) and NYN-Y453F-HLA-A24 (ID 8ZV9). NYN peptide (magenta), NYN-Y453F peptide (cyan), HLA-A24 (grey). **(E)** Comparison of unbound NYN-HLA-A24 (ID 7F4W) and TCR-bound TCR^NYN-I^-NYN-HLA-A24 (ID 8YE4) peptide presentation. The van der Waals and hydrogen bonds between P6-Tyr and TCR^NYN^ are blue and black dashes, respectively. **(F)** NYNYLYRLF and NYNYL**F**RLF P6 residues shown as magenta and cyan sticks, respectively. HLA-A24 shown as grey cartoon. TCRNYN-I CDR1α, CDR3α and CDR3β loops shown as red, yellow and green sticks, respectively. Hydrogen bonds are shown in black dashes. Van der Waals contacts are shown as blue dashes. **(G)** Structural rearrangements in RLQ-HLAs resulting from the T1006I mutation. RLQ-HLA-A2 (TCR RLQ7-HLA-A2, ID 8GOM), RLQ-T1006I-HLA-A2 (TCR RLQ7-T1006I-HLA-A2, ID 8GON), RLQ peptide (magenta), RLQ-T1006I peptide (cyan), HLA-A2 (grey). **(H)** Interactions between CDR1α of RLQ7 (red) and the RLQ peptide (magenta). Yellow sphere indicates an interfacial water molecule. Water-mediated hydrogen bonds are yellow dashed lines. Hydrogen bonds are black dashed lines. **(I)** Interactions between CDR1α of RLQ7 (red) and the RLQ-T1006I peptide (cyan). Hydrogen bonds are represented by black dashed.

Mutations within the epitope may disturb or change the peptide-dependent TCR contacts (29, 149). For the dominant YLQ epitopes, the original YLQ peptide forms six intra-peptide bonds to stabilize TCR binding, while the mutant YLQ-P272L-HLA-A2 (YLQ**L**RTFLL) structure exhibits fewer internal contacts (**Figure 5A**). The P5 arginine of the peptide underwent a significant conformational shift during the binding of TCR YLQ36 (**Figure 5B**). Superimposed structures of YLQ-P272L-HLA-A2 and YLQ36-YLQ-HLA2 complex revealed that the leucine of YLQ-P272L might protrude within 1˚ of the YLQ36 CDR3α loop, creating a steric hindrance between them (**Figure 5C**). This steric hindrance could potentially jeopardize the interaction between CDR3α and YLQ peptide, resulting in the loss of YLQ36 T cell recognition. Similarly, the spik-Y453F mutation within the NYN epitope is another dominant mutation that triggers HLA-A24-restricted CTL immune escape (91). In order to explore the escape molecular mechanism, we determined the crystal structures of original NYN-HLA-A24 (NYNYLYRLF) (92), mutant NYN-Y453F-HLA-A24 (NYNYL**F**RLF), and a ternary structure of TCR^NYN-I^-NYN-HLA-A24 (149). Structural analysis showed that after mutation or TCR^NYN-I^ binding, the conformation of the NYN peptide changed significantly, especially P4-Tyr, P5-Leu, and P6-Tyr/Phe (**Figures 5D, E**). The hydrophobic phenylalanine in the SARS-CoV-2 variants may disrupt contact network of the original tyrosine with TCR^NYN-I^, suggesting that despite competent presentation by HLA, the mutant Y453F peptide failed to establish a stable TCR-pHLA ternary complex due to reduced peptide: TCR contacts(**Figure 5F**). Unlike the P272L and Y453F dominant mutations, the RLQ-T1006I mutation was found to be tolerated in HLA-A24-restricted CTL activation (28, 30, 151). The T1006I substitution leads to a structural rearrangement of the HLA peptide-binding groove and alters the conformation of peptide residues P3-Gln and P6-Gln (**Figure 5G**). TCR RLQ7 CDR1α engages the N-terminal region of the original RLQ peptide via two direct and three water-mediated hydrogen bonds (**Figure 5H**), while TCR RLQ7 forms two new compensating hydrogen bonds with mutant RLQ-T1006I peptide (Asp31α with P4-Ser, Asp31α with P5-Leu) and an additional hydrogen bond with HLA-A2 (Glu29α with Arg66-HLA-A2) (**Figure 5I**). Similar stabilities of the two complexes may provide a reasonable explanation for the limited CTL immune escape.

Disruption or inhibition of antigen processing by mutation is a third mechanism of T cell immune escape. The components of the antigen processing pathway have preferences for their optimal amino acid sequences (152). It has been demonstrated in HIV-related studies that differences in amino acid sequence in the region flanking the epitope impaired the intracellular processing and presentation of epitope (153, 154). In the context of SARS-CoV-2 infection, a study proposed that variants may disrupt the HLA-I antigen presentation pathway by depleting proteasomes and altering the activity of ubiquitination enzymes, thereby preventing infected cells from presenting antigen proteins (148). A deeper understanding of the mechanisms by which SARS-CoV-2 evades host immune surveillance through the ubiquitin-proteasome system and HLA-I presentation warrants further investigation.

## 6 Conclusion

T cells are the backbone of the immune system and play a crucial role in the progression of COVID-19. HLA allele-related studies are essential for assessing the role of HLA in the immune response against SARS-CoV-2. Although some studies have demonstrated that HLA alleles were associated with differential susceptibility, these results were not consistent across studies. The lack of large-scale HLA typing in samples has limited the scope of research, with most studies involving sample sizes fewer than 190. Future large-scale studies are needed to provide a comprehensive understanding of the association between HLA genotypes and the evolving SARS-CoV-2 variants, thus improving our understanding of the association between antigen presentation and disease progression. Moreover, T cells carry a natural “barcode” sequence in their variable TCR region, specifically in the CDR3 component. Gene mutations and recombination endow the TCR repertoires great diversity and poly-clonality. Studies related to TCR-seq provide evidence for the close relationship between TCR diversity and anti-viral immunity. In the context of viral infection, the preferential selection of T cell clones narrows the TCR repertoire for antigen selection. Nevertheless, the full influence of SARS-CoV-2 on the TCR repertoire remains to be evaluated.

Although the characteristics of HLA polymorphisms and TCR repertoire in SARS-CoV-2 infection have been initially elucidated, the emergence of immune evasion variants complicates a comprehensive understanding of these relationships. Since the onset of the pandemic, SARS-CoV-2 has continued to evolve and adapt to its host, remaining a relatively new coronavirus. These variants are more prone to cause immune escape and vaccine escape. Studying the mechanisms of immune and vaccine escape remains a significant challenge. There is a need to conduct ongoing assessment of vaccine efficacy against these evolving variants, which may contribute to elucidate the drivers of the spread and evolutionary success of circulating variants. Addressing these unresolved issues promptly is crucial to ending the current pandemic and preparing for potential future ones.

## References

[1] Zhu N Zhang D Wang W Li X Yang B Song J. A novel coronavirus from patients with pneumonia in China, 2019. New Engl J Med. (2020) 382:727–33. doi: 10.1056/NEJMoa2001017 PMC7092803 31978945

[2] Wu Z McGoogan JM. Characteristics of and important lessons from the coronavirus disease 2019 (COVID-19) outbreak in China: summary of a report of 72 314 cases from the chinese center for disease control and prevention. JAMA. (2020) 323:1239–42. doi: 10.1001/jama.2020.2648 32091533

[3] Grasselli G Zangrillo A Zanella A Antonelli M Cabrini L Castelli A. Baseline characteristics and outcomes of 1591 patients infected with SARS-CoV-2 admitted to ICUs of the Lombardy Region, Italy. Jama. (2020) 323:1574–81. doi: 10.1001/jama.2020.5394 PMC7136855 32250385

[4] Rydyznski Moderbacher C Ramirez SI Dan JM Grifoni A Hastie KM Weiskopf D. Antigen-specific adaptive immunity to SARS-coV-2 in acute COVID-19 and associations with age and disease severity. Cell. (2020) 183:996–1012.e19. doi: 10.1016/j.cell.2020.09.038 33010815 PMC7494270

[5] Sette A Crotty S. Adaptive immunity to SARS-coV-2 and COVID-19. Cell. (2021) 184:861–80. doi: 10.1016/j.cell.2021.01.007 PMC7803150 33497610

[6] Robbiani DF Gaebler C Muecksch F Lorenzi JCC Wang Z Cho A. Convergent antibody responses to SARS-CoV-2 in convalescent individuals. Nature. (2020) 584:437–42. doi: 10.1038/s41586-020-2456-9 PMC7442695 32555388

[7] Klein SL Pekosz A Park H-S Ursin RL Shapiro JR Benner SE. Sex, age, and hospitalization drive antibody responses in a COVID-19 convalescent plasma donor population. J Clin Invest. (2020) 130:6141–50. doi: 10.1172/JCI142004 PMC7598041 32764200

[8] Long Q-X Tang X-J Shi Q-L Li Q Deng H-J Yuan J. Clinical and immunological assessment of asymptomatic SARS-CoV-2 infections. Nat Med. (2020) 26:1200–4. doi: 10.1038/s41591-020-0965-6 32555424

[9] Braun J Loyal L Frentsch M Wendisch D Georg P Kurth F. SARS-CoV-2-reactive T cells in healthy donors and patients with COVID-19. Nature. (2020) 587:270–4. doi: 10.1038/s41586-020-2598-9 32726801

[10] Grifoni A Weiskopf D Ramirez SI Mateus J Dan JM Moderbacher CR. Targets of T cell responses to SARS-coV-2 coronavirus in humans with COVID-19 disease and unexposed individuals. Cell. (2020) 181:1489–1501 e15. doi: 10.1016/j.cell.2020.05.015 32473127 PMC7237901

[11] Le Bert N Tan AT Kunasegaran K Tham CYL Hafezi M Chia A. SARS-CoV-2-specific T cell immunity in cases of COVID-19 and SARS, and uninfected controls. Nature. (2020) 584:457–62. doi: 10.1038/s41586-020-2550-z 32668444

[12] Peng Y Mentzer AJ Liu G Yao X Yin Z Dong D. Broad and strong memory CD4(+) and CD8(+) T cells induced by SARS-CoV-2 in UK convalescent individuals following COVID-19. Nat Immunol. (2020) 21:1336–45. doi: 10.1038/s41590-020-0782-6 PMC7611020 32887977

[13] Sekine T Perez-Potti A Rivera-Ballesteros O Stralin K Gorin JB Olsson A. Robust T cell immunity in convalescent individuals with asymptomatic or mild COVID-19. Cell. (2020) 183:158–168 e14. doi: 10.1016/j.cell.2020.08.017 32979941 PMC7427556

[14] Tang F Quan Y Xin Z-T Wrammert J Ma M-J Lv H. Lack of peripheral memory B cell responses in recovered patients with severe acute respiratory syndrome: A six-year follow-up study. J Immunol. (2011) 186:7264–8. doi: 10.4049/jimmunol.0903490 21576510

[15] Ng O-W Chia A Tan AT Jadi RS Leong HN Bertoletti A. Memory T cell responses targeting the SARS coronavirus persist up to 11 years post-infection. Vaccine. (2016) 34:2008–14. doi: 10.1016/j.vaccine.2016.02.063 PMC7115611 26954467

[16] Soresina A Moratto D Chiarini M Paolillo C Baresi G Focà E. Two X-linked agammaglobulinemia patients develop pneumonia as COVID-19 manifestation but recover. Pediatr Allergy Immunol. (2020) 31:565–9. doi: 10.1111/pai.13263 PMC7264678 32319118

[17] Thevarajan I Nguyen THO Koutsakos M Druce J Caly L van de Sandt CE. Breadth of concomitant immune responses prior to patient recovery: a case report of non-severe COVID-19. Nat Med. (2020) 26:453–5. doi: 10.1038/s41591-020-0819-2 PMC7095036 32284614

[18] Zhang X Tan Y Ling Y Lu G Liu F Yi Z. Viral and host factors related to the clinical outcome of COVID-19. Nature. (2020) 583:437–40. doi: 10.1038/s41586-020-2355-0 32434211

[19] Minervina AA Komech EA Titov A Bensouda Koraichi M Rosati E Mamedov IZ. Longitudinal high-throughput TCR repertoire profiling reveals the dynamics of T-cell memory formation after mild COVID-19 infection. Elife. (2021) 10:e63502. doi: 10.7554/eLife.63502 33399535 PMC7806265

[20] Agerer B Koblischke M Gudipati V Montaño-Gutierrez LF Smyth M Popa A. SARS-CoV-2 mutations in MHC-I-restricted epitopes evade CD8+ T cell responses. Sci Immunol. (2021) 6:eabg6461. doi: 10.1126/sciimmunol.abg6461 33664060 PMC8224398

[21] Dan JM Mateus J Kato Y Hastie KM Yu ED Faliti CE. Immunological memory to SARS-CoV-2 assessed for up to 8 months after infection. Science. (2021) 371:eabf4063. doi: 10.1126/science.abf4063 33408181 PMC7919858

[22] Zuo J Dowell AC Pearce H Verma K Long HM Begum J. Robust SARS-CoV-2-specific T cell immunity is maintained at 6 months following primary infection. Nat Immunol. (2021) 22:620–6. doi: 10.1038/s41590-021-00902-8 PMC7610739 33674800

[23] Sahin U Muik A Vogler I Derhovanessian E Kranz LM Vormehr M. BNT162b2 vaccine induces neutralizing antibodies and poly-specific T cells in humans. Nature. (2021) 595:572–7. doi: 10.1038/s41586-021-03653-6 34044428

[24] Weiskopf D Schmitz KS Raadsen MP Grifoni A Okba NMA Endeman H. Phenotype and kinetics of SARS-CoV-2-specific T cells in COVID-19 patients with acute respiratory distress syndrome. Sci Immunol. (2020) 5:eabd2071. doi: 10.1126/sciimmunol.abd2071 32591408 PMC7319493

[25] Marsh SGE Parham P Barber LD. Evolution and anthropology of HLA. The HLA Facts Book. London: Academic Press (2000), 73–83.

[26] Falk K Rötzschke O Stevanović S Jung G Rammensee H-G. Allele-specific motifs revealed by sequencing of self-peptides eluted from MHC molecules. 1991. J Immunol (Baltimore Md.: 1950). (2006) 177:2741–7. doi: 101038/351290a0 16920906

[27] Rammensee H-G Falk K Rötzschke O. MHC molecules as peptide receptors. Curr Opin Immunol. (1993) 5:35–44. doi: 10.1016/0952-7915(93)90078-7 7680871

[28] Wu D Kolesnikov A Yin R Guest JD Gowthaman R Shmelev A. Structural assessment of HLA-A2-restricted SARS-CoV-2 spike epitopes recognized by public and private T-cell receptors. Nat Commun. (2022) 13:19–9. doi: 10.1038/s41467-021-27669-8 PMC8748687 35013235

[29] Dolton G Rius C Hasan MS Wall A Szomolay B Behiry E. Emergence of immune escape at dominant SARS-CoV-2 killer T cell epitope. Cell. (2022) 185:2936–2951 e19. doi: 10.1016/j.cell.2022.07.002 35931021 PMC9279490

[30] Swaminathan S Lineburg KE Panikkar A Raju J Murdolo LD Szeto C. Ablation of CD8(+) T cell recognition of an immunodominant epitope in SARS-CoV-2 Omicron variants BA.1, BA.2 and BA.3. Nat Commun. (2022) 13:6387. doi: 10.1038/s41467-022-34180-1 36302758 PMC9607807

[31] Kombe Kombe AJ Biteghe FAN Ndoutoume ZN Jin T. CD8(+) T-cell immune escape by SARS-CoV-2 variants of concern. Front Immunol. (2022) 13:962079. doi: 10.3389/fimmu.2022.962079 36389664 PMC9647062

[32] Quiñones-Parra S Grant E Loh L Nguyen TH Campbell K-A Tong SY. Preexisting CD8+ T-cell immunity to the H7N9 influenza A virus varies across ethnicities. Proc Natl Acad Sci. (2014) 111:1049–54. doi: 101016/s0140-6736(70)90244-8 10.1073/pnas.1322229111 PMC3903243 24395804

[33] Hertz T Oshansky CM Roddam PL DeVincenzo JP Caniza MA Jojic N. HLA targeting efficiency correlates with human T-cell response magnitude and with mortality from influenza A infection. Proc Natl Acad Sci. (2013) 110:13492–7. doi: 10.1073/pnas.1221555110 PMC3746844 23878211

[34] Kenney AD Dowdle JA Bozzacco L McMichael TM St Gelais C Panfil AR. Human genetic determinants of viral diseases. Annu Rev Genet. (2017) 51:241–63. doi: 10.1146/annurev-genet-120116-023425 PMC6038703 28853921

[35] Carter-Timofte ME Jørgensen SE Freytag MR Thomsen MM Brinck Andersen N-S Al-Mousawi A. Deciphering the role of host genetics in susceptibility to severe COVID-19. Front Immunol. (2020) 11:1606. doi: 10.3389/fimmu.2020.01606 32695122 PMC7338588

[36] JL A. Study of leucocyte phenotypes in Hodgkins’ disease. Lancet. (1967) 2(7676):771–2. 10.1016/s0140-6736(70)90244-8 4195991

[37] Lunardi LW. Bragatte, M.A.d.S. & Vieira, G.F. The influence of HLA/HIV genetics on the occurrence of elite controllers and a need for therapeutics geotargeting view. Braz J Infect Dis. (2021) 25:101619. doi: 10.1016/j.bjid.2021.101619 34562387 PMC9392165

[38] Carrington M Nelson GW Martin MP Kissner T Vlahov D Goedert JJ. HLA and HIV-1: heterozygote advantage and B*35-cw*04 disadvantage. Science. (1999) 283:1748–52. doi: 10.1126/science.283.5408.1748 10073943

[39] Apps R Qi Y Carlson JM Chen H Gao X Thomas R. Influence of HLA-C expression level on HIV control. Science. (2013) 340:87–91. doi: 10.1126/science.1232685 23559252 PMC3784322

[40] Blackwell Jenefer M Jamieson Sarra E Burgner D. HLA and infectious diseases. Clin Microbiol Rev. (2009) 22:370–85. doi: 10.1128/CMR.00048-08 PMC2668228 19366919

[41] Niemi MEK Karjalainen J Liao RG Neale BM Daly M Ganna A. Mapping the human genetic architecture of COVID-19. Nature. (2021) 600:472–7. doi: 10.1038/s41586-021-03767-x PMC8674144 34237774

[42] Ben Shachar S Barda N Manor S Israeli S Dagan N Carmi S. MHC haplotyping of SARS-CoV-2 patients: HLA subtypes are not associated with the presence and severity of COVID-19 in the Israeli population. J Clin Immunol. (2021) 41:1154–61. doi: 10.1007/s10875-021-01071-x PMC8164405 34050837

[43] Shkurnikov M Nersisyan S Jankevic T Galatenko A Gordeev I Vechorko V. Association of HLA class I genotypes with severity of coronavirus disease-19. Front Immunol. (2021) 12. doi: 10.3389/fimmu.2021.641900 PMC7959787 33732261

[44] Anzurez A Naka I Miki S Nakayama-Hosoya K Isshiki M Watanabe Y. Association of HLA-*09:01 with severe COVID-19. HLA. (2021) 98:37–42. doi: 10.1111/tan.14256 33734601 PMC8251239

[45] Yung Y-L Cheng C-K Chan H-Y Xia JT Lau K-M Wong RSM. Association of HLA-B22 serotype with SARS-CoV-2 susceptibility in Hong Kong Chinese patients. HLA. (2021) 97:127–32. doi: 10.1111/tan.14135 PMC7898481 33179437

[46] Wang W Zhang W Zhang J He J Zhu F. Distribution of HLA allele frequencies in 82 Chinese individuals with coronavirus disease-2019 (COVID-19). Hla. (2020) 96:194–6. doi: 10.1111/tan.13941 PMC7276866 32424945

[47] Null N. Genomewide association study of severe covid-19 with respiratory failure. New Engl J Med. (2020) 383:1522–34. doi: 10.1056/NEJMoa2020283 PMC7315890 32558485

[48] Novelli A Andreani M Biancolella M Liberatoscioli L Passarelli C Colona VL. HLA allele frequencies and susceptibility to COVID-19 in a group of 99 Italian patients. Hla. (2020) 96:610–4. doi: 10.1111/tan.14047 PMC7461491 32827207

[49] Amoroso A Magistroni P Vespasiano F Bella A Bellino S Puoti F. HLA and AB0 polymorphisms may influence SARS-coV-2 infection and COVID-19 severity. Transplantation. (2021) 105:193–200. doi: 10.1097/TP.0000000000003507 33141807

[50] Lorente L Martín MM Franco A Barrios Y Cáceres JJ Solé-Violán J. HLA genetic polymorphisms and prognosis of patients with COVID-19. Med Intensiva. (2021) 45:96–103. doi: 10.1016/j.medin.2020.08.004 PMC7474921 38620408

[51] Wang F Huang S Gao R Zhou Y Lai C Li Z. Initial whole-genome sequencing and analysis of the host genetic contribution to COVID-19 severity and susceptibility. Cell Discovery. (2020) 6:83. doi: 10.1038/s41421-020-00231-4 33298875 PMC7653987

[52] Leite M Gonzalez-Galarza FF Silva B Middleton D Santos E. Predictive immunogenetic markers in COVID-19. Hum Immunol. (2021) 82:247–54. doi: 10.1016/j.humimm.2021.01.008 PMC7817393 33546902

[53] Pisanti S Deelen J Gallina AM Caputo M Citro M Abate M. Correlation of the two most frequent HLA haplotypes in the Italian population to the differential regional incidence of Covid-19. J Trans Med. (2020) 18:1–16. doi: 10.1186/s12967-020-02515-5 PMC7491019 32933522

[54] Bacher P Rosati E Esser D Martini GR Saggau C Schiminsky E. Low-avidity CD4+ T cell responses to SARS-CoV-2 in unexposed individuals and humans with severe COVID-19. Immunity. (2020) 53:1258–1271. e5. doi: 10.1016/j.immuni.2020.11.016 33296686 PMC7689350

[55] Khor S-S Omae Y Nishida N Sugiyama M Kinoshita N Suzuki T. HLA-A* 11: 01: 01: 01, HLA-C* 12: 02: 02: 01-HLA-B* 52: 01: 02: 02, age and sex are associated with severity of Japanese COVID-19 with respiratory failure. Front Immunol. (2021) 12:658570. doi: 10.3389/fimmu.2021.658570 33968060 PMC8100314

[56] Weiner J Suwalski P Holtgrewe M Rakitko A Thibeault C Mueller M. Increased risk of severe clinical course of COVID-19 in carriers of HLA-C* 04: 01. EClinicalMedicine. (2021) 40:101099. doi: 10.1016/j.eclinm.2021.101099 34490415 PMC8410317

[57] Vietzen H Zoufaly A Traugott M Aberle J Aberle SW Puchhammer-Stöckl E. Deletion of the NKG2C receptor encoding KLRC2 gene and HLA-E variants are risk factors for severe COVID-19. Genet Med. (2021) 23:963–7. doi: 10.1038/s41436-020-01077-7 PMC7835668 33500568

[58] Schindler E Dribus M Duffy BF Hock K Farnsworth CW Gragert L. HLA genetic polymorphism in patients with Coronavirus Disease 2019 in Midwestern United States. Hla. (2021) 98:370–9. doi: 10.1111/tan.14387 PMC8429120 34338446

[59] Nguyen A David JK Maden SK Wood MA Weeder BR Nellore A. Human leukocyte antigen susceptibility map for severe acute respiratory syndrome coronavirus 2. J Virol. (2020) 94:e00510-20. doi: 10.1128/jvi.00510-20 32303592 PMC7307149

[60] Augusto DG Murdolo LD Chatzileontiadou DSM Sabatino JJ Yusufali T Peyser ND. A common allele of HLA is associated with asymptomatic SARS-CoV-2 infection. Nature. (2023) 620:128–36. doi: 10.1038/s41586-023-06331-x PMC10396966 37468623

[61] Triunfol M. HLA-B*15:01 allele and asymptomatic SARS-CoV-2 infection. Lancet Respir Med. (2023) 11:e83. doi: 10.1016/S2213-2600(23)00295-3 37549678

[62] Littera R Campagna M Deidda S Angioni G Cipri S Melis M. Human leukocyte antigen complex and other immunogenetic and clinical factors influence susceptibility or protection to SARS-CoV-2 infection and severity of the disease course. The Sardinian experience. Front Immunol. (2020) 11:605688. doi: 10.3389/fimmu.2020.605688 33343579 PMC7746644

[63] Lin M Tseng H-K Trejaut JA Lee H-L Loo J-H Chu C-C. Association of HLA class I with severe acute respiratory syndrome coronavirus infection. BMC Med Genet. (2003) 4:9–9. doi: 10.1186/1471-2350-4-9 12969506 PMC212558

[64] Grifoni A Sidney J Vita R Peters B Crotty S Weiskopf D. SARS-CoV-2 human T cell epitopes: Adaptive immune response against COVID-19. Cell Host Microbe. (2021) 29:1076–92. doi: 10.1016/j.chom.2021.05.010 PMC8139264 34237248

[65] Immune Epitope Database (IEDB). (2023). Available online at: http://www.iedb.org/. (accessed April 25, 2024).

[66] Ferretti AP Kula T Wang Y Nguyen DMV Weinheimer A Dunlap GS. Unbiased screens show CD8(+) T cells of COVID-19 patients recognize shared epitopes in SARS-coV-2 that largely reside outside the spike protein. Immunity. (2020) 53:1095–1107 e3. doi: 10.1016/j.immuni.2020.10.006 33128877 PMC7574860

[67] Nelde A Bilich T Heitmann JS Maringer Y Salih HR Roerden M. SARS-CoV-2-derived peptides define heterologous and COVID-19-induced T cell recognition. Nat Immunol. (2021) 22:74–85. doi: 10.1038/s41590-020-00808-x 32999467

[68] Tarke A Sidney J Kidd CK Dan JM Ramirez SI Yu ED. Comprehensive analysis of T cell immunodominance and immunoprevalence of SARS-CoV-2 epitopes in COVID-19 cases. Cell Rep Med. (2021) 2:100204. doi: 10.1016/j.xcrm.2021.100204 33521695 PMC7837622

[69] Keller MD Harris KM Jensen-Wachspress MA Kankate VV Lang H Lazarski CA. SARS-CoV-2–specific T cells are rapidly expanded for therapeutic use and target conserved regions of the membrane protein. Blood J Am Soc Hematol. (2020) 136:2905–17. doi: 10.1182/blood.2020008488 PMC7746091 33331927

[70] Nielsen SS Vibholm LK Monrad I Olesen R Frattari GS Pahus MH. SARS-CoV-2 elicits robust adaptive immune responses regardless of disease severity. EBioMedicine. (2021) 68:103410. doi: 10.1016/j.ebiom.2021.103410 34098342 PMC8176920

[71] Shomuradova AS Vagida MS Sheetikov SA Zornikova KV Kiryukhin D Titov A. SARS-coV-2 epitopes are recognized by a public and diverse repertoire of human T cell receptors. Immunity. (2020) 53:1245–1257.e5. doi: 10.1016/j.immuni.2020.11.004 33326767 PMC7664363

[72] Alter G Yu J Liu J Chandrashekar A Borducchi EN Tostanoski LH. Immunogenicity of Ad26.COV2.S vaccine against SARS-CoV-2 variants in humans. Nature. (2021) 596:268–72. doi: 10.1038/s41586-021-03681-2 PMC8357629 34107529

[73] Rha MS Jeong HW Ko JH Choi SJ Seo IH Lee JS. PD-1-expressing SARS-coV-2-specific CD8(+) T cells are not exhausted, but functional in patients with COVID-19. Immunity. (2021) 54:44–52 e3. doi: 10.1016/j.immuni.2020.12.002 33338412 PMC7834198

[74] Sahin U Muik A Vogler I Derhovanessian E Kranz LM Vormehr M. BNT162b2 induces SARS-CoV-2-neutralising antibodies and T cells in humans. MedRxiv. (2020) 2020:12. 09.20245175. doi: 101038/s41586-021-03653-6

[75] Saini SK Hersby DS Tamhane T Povlsen HR Hernandez SPA Nielsen M. SARS-CoV-2 genome-wide mapping of CD8 T cell recognition reveals strong immunodominance and substantial CD8 T cell activation in COVID-19 patients. Sci Immunol. (2020) 6(58):eabf7550. doi: 10.1101/2020.10.19.344911 PMC8139428 33853928

[76] Saini SK Hersby DS Tamhane T Povlsen HR Hernandez SPA Nielsen M. SARS-CoV-2 genome-wide T cell epitope mapping reveals immunodominance and substantial CD8(+) T cell activation in COVID-19 patients. Sci Immunol. (2021) 6:eabf7550. doi: 10.1126/sciimmunol.abf7550 33853928 PMC8139428

[77] Oberhardt V Luxenburger H Kemming J Schulien I Ciminski K Giese S. Rapid and stable mobilization of CD8(+) T cells by SARS-CoV-2 mRNA vaccine. Nature. (2021) 597:268–73. doi: 10.1038/s41586-021-03841-4 PMC8426185 34320609

[78] Nguyen TH Rowntree LC Allen LF Chua BY Kedzierski L Lim C. Robust SARS-CoV-2 T cell responses with common TCRαβ motifs toward COVID-19 vaccines in patients with hematological Malignancy impacting B cells. Cell Rep Med. (2023) 4:101017. doi: 10.1016/j.xcrm.2023.101017 37030296 PMC10040362

[79] Minervina AA Pogorelyy MV Kirk AM Allen EK Allison KJ Lin C-Y. Convergent epitope-specific T cell responses after SARS-CoV-2 infection and vaccination. Nat Immunol. (2021) 23(5):781–90. doi: 101038/s41590-022-01184-4

[80] Nguyen THO Rowntree LC Petersen J Chua BY Hensen L Kedzierski L. CD8(+) T cells specific for an immunodominant SARS-CoV-2 nucleocapsid epitope display high naive precursor frequency and TCR promiscuity. Immunity. (2021) 54:1066–1082 e5. doi: 10.1016/j.immuni.2021.04.009 33951417 PMC8049468

[81] Szeto C Nguyen AT Lobos CA Chatzileontiadou DSM Jayasinghe D Grant EJ. Molecular basis of a dominant SARS-coV-2 spike-derived epitope presented by HLA-A*02:01 recognised by a public TCR. Cells. (2021) 10:2646. doi: 10.3390/cells10102646 34685626 PMC8534114

[82] Rowntree LC Nguyen THO Kedzierski L Neeland MR Petersen J Crawford JC. SARS-CoV-2-specific T cell memory with common TCRalphabeta motifs is established in unvaccinated children who seroconvert after infection. Immunity. (2022) 55:1299–1315 e4. doi: 10.1016/j.immuni.2022.06.003 35750048 PMC9174177

[83] Rowntree LC Petersen J Juno JA Chaurasia P Wragg K Koutsakos M. SARS-CoV-2-specific CD8(+) T-cell responses and TCR signatures in the context of a prominent HLA-A*24:02 allomorph. Immunol Cell Biol. (2021) 99:990–1000. doi: 10.1111/imcb.12482 34086357 PMC8242669

[84] Francis JA-O Leistritz-Edwards DA-O Dunn AA-O Tarr C Lehman JA-O Dempsey C. Allelic variation in class I HLA determines CD8(+) T cell repertoire shape and cross-reactive memory responses to SARS-CoV-2. Sci Immunol. (2021) 7(67):eabk3070. doi: 101126/sciimmunolabk3070 10.1126/sciimmunol.abk3070 PMC9017864 34793243

[85] Lineburg KE Grant EJ Swaminathan S Chatzileontiadou DS Szeto C Sloane H. CD8+ T cells specific for an immunodominant SARS-CoV-2 nucleocapsid epitope cross-react with selective seasonal coronaviruses. Immunity. (2021) 54:1055–1065. e5. doi: 10.1016/j.immuni.2021.04.006 33945786 PMC8043652

[86] Hu C Shen M Han X Chen Q Li L Chen S. Identification of cross-reactive CD8(+) T cell receptors with high functional avidity to a SARS-CoV-2 immunodominant epitope and its natural mutant variants. Genes Dis. (2022) 9:216–29. doi: 10.1016/j.gendis.2021.05.006 PMC8240504 34222571

[87] Braciale TJ Sun J Kim TS. Regulating the adaptive immune response to respiratory virus infection. Nat Rev Immunol. (2012) 12:295–305. doi: 10.1038/nri3166 22402670 PMC3364025

[88] de Silva TI Liu G Lindsey BB Dong D Moore SC Hsu NS. The impact of viral mutations on recognition by SARS-CoV-2 specific T cells. iScience. (2021) 24:103353. doi: 10.1016/j.isci.2021.103353 34729465 PMC8552693

[89] Qiu C Xiao C Wang Z Zhu G Mao L Chen X. CD8(+) T-cell epitope variations suggest a potential antigen HLA-A2 binding deficiency for spike protein of SARS-coV-2. Front Immunol. (2021) 12:764949. doi: 103389/fimmu2021764949 35116022 10.3389/fimmu.2021.764949 PMC8804355

[90] Wilkinson SAJ Richter A Casey A Osman H Mirza JD Stockton J. Recurrent SARS-CoV-2 mutations in immunodeficient patients. Virus Evol. (2022) 8:veac050–veac050. doi: 10.1093/ve/veac050 35996593 PMC9384748

[91] Motozono C Toyoda M Zahradnik J Saito A Nasser H Tan TS. SARS-CoV-2 spike L452R variant evades cellular immunity and increases infectivity. Cell Host Microbe. (2021) 29:1124–1136 e11. doi: 10.1016/j.chom.2021.06.006 34171266 PMC8205251

[92] Zhang H Deng S Ren L Zheng P Hu X Jin T. Profiling CD8+ T cell epitopes of COVID-19 convalescents reveals reduced cellular immune responses to SARS-CoV-2 variants. Cell Rep. (2021) 36:109708. doi: 10.1016/j.celrep.2021.109708 34506741 PMC8390359

[93] Hansen TH Bouvier M. MHC class I antigen presentation: learning from viral evasion strategies. Nat Rev Immunol. (2009) 9:503–13. doi: 10.1038/nri2575 19498380

[94] Quigley MF Greenaway HY Venturi V Lindsay R Quinn KM Seder RA. Convergent recombination shapes the clonotypic landscape of the naive T-cell repertoire. Proc Natl Acad Sci. (2010) 107:19414–9. doi: 10.1073/pnas.1010586107 PMC2984183 20974936

[95] Huang H Sikora MJ Islam S Chowdhury RR Chien Y-h Scriba TJ. Select sequencing of clonally expanded CD8+ T cells reveals limits to clonal expansion. Proc Natl Acad Sci. (2019) 116:8995–9001. doi: 10.1073/pnas.1902649116 30992377 PMC6500157

[96] Britanova OV Shugay M Merzlyak EM Staroverov DB Putintseva EV Turchaninova MA. Dynamics of individual T cell repertoires: from cord blood to centenarians. J Immunol. (2016) 196:5005–13. doi: 10.4049/jimmunol.1600005 27183615

[97] Gutierrez L Beckford J Alachkar H. Deciphering the TCR repertoire to solve the COVID-19 mystery. Trends Pharmacol Sci. (2020) 41:518–30. doi: 10.1016/j.tips.2020.06.001 PMC7305739 32576386

[98] Nguyen TH Sant S Bird NL Grant EJ Clemens EB Koutsakos M. Perturbed CD8+ T cell immunity across universal influenza epitopes in the elderly. J Leukocyte Biol. (2018) 103:321–39. doi: 10.1189/jlb.5MA0517-207R 28928269

[99] Gil A Yassai MB Naumov YN Selin LK. Narrowing of human influenza A virus-specific T cell receptor α and β repertoires with increasing age. J Virol. (2015) 89:4102–16. doi: 10.1128/JVI.03020-14 PMC4442365 25609818

[100] Zhou F Yu T Du R Fan G Liu Y Liu Z. Clinical course and risk factors for mortality of adult inpatients with COVID-19 in Wuhan, China: a retrospective cohort study. Lancet. (2020) 395:1054–62. doi: 10.1016/S0140-6736(20)30566-3 PMC7270627 32171076

[101] Ruan Q Yang K Wang W Jiang L Song J. Clinical predictors of mortality due to COVID-19 based on an analysis of data of 150 patients from Wuhan, China. Intensive Care Med. (2020) 46:846–8. doi: 10.1007/s00134-020-05991-x PMC7080116 32125452

[102] Sharon E Sibener LV Battle A Fraser HB Garcia KC Pritchard JK. Genetic variation in MHC proteins is associated with T cell receptor expression biases. Nat Genet. (2016) 48:995–1002. doi: 10.1038/ng.3625 27479906 PMC5010864

[103] Falfán-Valencia R Narayanankutty A Reséndiz-Hernández JM Pérez-Rubio G Ramírez-Venegas A Nava-Quiroz KJ. An increased frequency in HLA class I alleles and haplotypes suggests genetic susceptibility to influenza A (H1N1) 2009 pandemic: A case-control study. J Immunol Res. (2018) 2018:3174868. doi: 10.1155/2018/3174868 29682588 PMC5845504

[104] Thomas MS Rachel MG Mark K Damon HM Edward JO Ruth T. Magnitude and dynamics of the T-cell response to SARS-coV-2 infection at both individual and population levels. medRxiv. (2020) 17:2020073120165647. doi: 101101/2020073120165647

[105] Adamo S Michler J Zurbuchen Y Cervia C Taeschler P Raeber ME. Signature of long-lived memory CD8(+) T cells in acute SARS-CoV-2 infection. Nature. (2022) 602:148–55. doi: 10.1038/s41586-021-04280-x PMC8810382 34875673

[106] Luo L Liang W Pang J Xu G Chen Y Guo X. Dynamics of TCR repertoire and T cell function in COVID-19 convalescent individuals. Cell Discovery. (2021) 7:89. doi: 10.1038/s41421-021-00321-x 34580278 PMC8476510

[107] Bilich T Nelde A Heitmann JS Maringer Y Roerden M Bauer J. T cell and antibody kinetics delineate SARS-CoV-2 peptides mediating long-term immune responses in COVID-19 convalescent individuals. Sci Trans Med. (2021) 13:eabf7517. doi: 10.1126/scitranslmed.abf7517 PMC8128286 33723016

[108] Cohen KW Linderman SL Moodie Z Czartoski J Lai L Mantus G. Longitudinal analysis shows durable and broad immune memory after SARS-CoV-2 infection with persisting antibody responses and memory B and T cells. Cell Rep Med. (2021) 2:100354. doi: 10.1016/j.xcrm.2021.100354 34250512 PMC8253687

[109] Zornikova KV Khmelevskaya A Sheetikov SA Kiryukhin DO Shcherbakova OV Titov A. Clonal diversity predicts persistence of SARS-CoV-2 epitope-specific T-cell response. Commun Biol. (2022) 5:1351. doi: 10.1038/s42003-022-04250-7 36494499 PMC9734123

[110] Miconnet I Marrau A Farina A Taffé P Vigano S Harari . Large TCR diversity of virus-specific CD8 T cells provides the mechanistic basis for massive TCR renewal after antigen exposure. J Immunol. (2011) 186:7039–49. doi: 10.4049/jimmunol.1003309 21555537

[111] Dash P Fiore-Gartland AJ Hertz T Wang GC Sharma S Souquette A. Quantifiable predictive features define epitope-specific T cell receptor repertoires. Nature. (2017) 547:89–93. doi: 10.1038/nature22383 28636592 PMC5616171

[112] Price DA Brenchley JM Ruff LE Betts MR Hill BJ Roederer M. Avidity for antigen shapes clonal dominance in CD8+ T cell populations specific for persistent DNA viruses. J Exp Med. (2005) 202:1349–61. doi: 10.1084/jem.20051357 PMC2212993 16287711

[113] Zehn D Lee SY Bevan MJ. Complete but curtailed T-cell response to very low-affinity antigen. Nature. (2009) 458:211–4. doi: 10.1038/nature07657 PMC2735344 19182777

[114] Sureshchandra S Lewis SA Doratt BM Jankeel A Ibraim IC Messaoudi I. Single-cell profiling of T and B cell repertoires following SARS-CoV-2 mRNA vaccine. JCI Insight. (2021) 6:e153201. doi: 10.1172/jci.insight.153201 34935643 PMC8783687

[115] Taus E Hofmann C Ibarrondo FJ Gong LS Hausner MA Fulcher JA. Persistent memory despite rapid contraction of circulating T Cell responses to SARS-CoV-2 mRNA vaccination. Front Immunol. (2023) 14. doi: 10.3389/fimmu.2023.1100594 PMC9968837 36860850

[116] Ford ES Mayer-Blackwell K Jing L Laing KJ Sholukh AM St Germain R. Repeated mRNA vaccination sequentially boosts SARS-CoV-2-specific CD8+ T cells in persons with previous COVID-19. Nat Immunol. (2024) 25:166–77. doi: 10.1038/s41590-023-01692-x PMC10981451 38057617

[117] Mayer-Blackwell K Ryu H Codd AS Parks KR MacMillan HR Cohen KW. mRNA vaccination boosts S-specific T cell memory and promotes expansion of CD45RAint TEMRA-like CD8+ T cells in COVID-19 recovered individuals. Cell Rep Med. (2023) 4:101149. doi: 10.1016/j.xcrm.2023.101149 37552991 PMC10439252

[118] Aoki H Kitabatake M Abe H Xu P Tsunoda M Shichino S. CD8+ T cell memory induced by successive SARS-CoV-2 mRNA vaccinations is characterized by shifts in clonal dominance. Cell Rep. (2024) 43:113887. doi: 10.1016/j.celrep.2024.113887 38458195

[119] Lang-Meli J Luxenburger H Wild K Karl V Oberhardt V Salimi Alizei E. SARS-CoV-2-specific T-cell epitope repertoire in convalescent and mRNA-vaccinated individuals. Nat Microbiol. (2022) 7:675–9. doi: 10.1038/s41564-022-01106-y PMC9064790 35484232

[120] Pantaleo G Demarest JF Soudeyns H Graziosi C Denis F Adelsberger JW. Major expansion of CD8+ T cells with a predominant Vβ usage during the primary immune response to HIV. Nature. (1994) 370:463–7. doi: 10.1038/370463a0 8047166

[121] Wang P Jin X Zhou W Luo M Xu Z Xu C. Comprehensive analysis of TCR repertoire in COVID-19 using single cell sequencing. Genomics. (2021) 113:456–62. doi: 10.1016/j.ygeno.2020.12.036 PMC7833309 33383142

[122] Bai H Ma J Mao W Zhang X Nie Y Hao J. Identification of TCR repertoires in asymptomatic COVID-19 patients by single-cell T-cell receptor sequencing. Blood Cells Molecules Dis. (2022) 97:102678. doi: 10.1016/j.bcmd.2022.102678 PMC9162783 35716403

[123] Sen K Datta S Ghosh A Jha A Ahad A Chatterjee S. Single-cell immunogenomic approach identified SARS-CoV-2 protective immune signatures in asymptomatic direct contacts of COVID-19 cases. Front Immunol. (2021) 12:733539. doi: 10.3389/fimmu.2021.733539 34899693 PMC8660575

[124] Cheng MH Zhang S Porritt RA Noval Rivas M Paschold L Willscher E. Superantigenic character of an insert unique to SARS-CoV-2 spike supported by skewed TCR repertoire in patients with hyperinflammation. Proc Natl Acad Sci. (2020) 117:25254–62. doi: 10.1073/pnas.2010722117 PMC7568239 32989130

[125] Sidhom J-W Baras AS. Deep learning identifies antigenic determinants of severe SARS-CoV-2 infection within T-cell repertoires. Sci Rep. (2021) 11:14275. doi: 10.1038/s41598-021-93608-8 34253751 PMC8275616

[126] Laydon DJ Bangham CRM Asquith B. Estimating T-cell repertoire diversity: limitations of classical estimators and a new approach. Philos Trans R Soc B: Biol Sci. (2015) 370:20140291. doi: 10.1098/rstb.2014.0291 PMC4528489 26150657

[127] Nikolich-Žugich J Slifka MK Messaoudi I. The many important facets of T-cell repertoire diversity. Nat Rev Immunol. (2004) 4:123–32. doi: 10.1038/nri1292 15040585

[128] Glanville J Huang H Nau A Hatton O Wagar LE Rubelt F. Identifying specificity groups in the T cell receptor repertoire. Nature. (2017) 547:94–8. doi: 10.1038/nature22976 PMC5794212 28636589

[129] Venturi V Price DA Douek DC Davenport MP. The molecular basis for public T-cell responses? Nat Rev Immunol. (2008) 8:231–8. doi: 101038/nri2260 10.1038/nri2260 18301425

[130] Chen H Ndhlovu ZM Liu D Porter LC Fang JW Darko S. TCR clonotypes modulate the protective effect of HLA class I molecules in HIV-1 infection. Nat Immunol. (2012) 13:691–700. doi: 10.1038/ni.2342 22683743 PMC3538851

[131] Almeida JR Sauce D Price DA Papagno L Shin SY Moris A. Antigen sensitivity is a major determinant of CD8+ T-cell polyfunctionality and HIV-suppressive activity. Blood. (2009) 113:6351–60. doi: 10.1182/blood-2009-02-206557 PMC2710928 19389882

[132] Qi Q Liu Y Cheng Y Glanville J Zhang D Lee J-Y. Diversity and clonal selection in the human T-cell repertoire. Proc Natl Acad Sci. (2014) 111:13139–44. doi: 10.1073/pnas.1409155111 PMC4246948 25157137

[133] Schulien I Kemming J Oberhardt V Wild K Seidel LM Killmer S. Characterization of pre-existing and induced SARS-CoV-2-specific CD8+ T cells. Nat Med. (2021) 27:78–85. doi: 10.1038/s41591-020-01143-2 33184509

[134] Minervina AA Pogorelyy MV Kirk AM Crawford JC Allen EK Chou C-H. SARS-CoV-2 antigen exposure history shapes phenotypes and specificity of memory CD8+ T cells. Nat Immunol. (2022) 23:781–90. doi: 10.1038/s41590-022-01184-4 PMC9106845 35383307

[135] Goncharov M Bagaev D Shcherbinin D Zvyagin I Bolotin D Thomas PG. VDJdb in the pandemic era: a compendium of T cell receptors specific for SARS-CoV-2. Nat Methods. (2022) 19:1017–9. doi: 10.1038/s41592-022-01578-0 35970936

[136] Pogorelyy MV Fedorova AD McLaren JE Ladell K Bagaev DV Eliseev AV. Exploring the pre-immune landscape of antigen-specific T cells. Genome Med. (2018) 10:68. doi: 10.1186/s13073-018-0577-7 30144804 PMC6109350

[137] Culshaw A Ladell K Gras S McLaren JE Miners KL Farenc C. Germline bias dictates cross-serotype reactivity in a common dengue-virus-specific CD8+ T cell response. Nat Immunol. (2017) 18:1228–37. doi: 10.1038/ni.3850 28945243

[138] Price DA West SM Betts MR Ruff LE Brenchley JM Ambrozak DR. T cell receptor recognition motifs govern immune escape patterns in acute SIV infection. Immunity. (2004) 21:793–803. doi: 10.1016/j.immuni.2004.10.010 15589168

[139] Mateus J Grifoni A Tarke A Sidney J Ramirez SI Dan JM. Selective and cross-reactive SARS-CoV-2 T cell epitopes in unexposed humans. Science. (2020) 370:89–94. doi: 10.1126/science.abd3871 32753554 PMC7574914

[140] Sette A Crotty S. Pre-existing immunity to SARS-CoV-2: the knowns and unknowns. Nat Rev Immunol. (2020) 20:457–8. doi: 10.1038/s41577-020-0389-z PMC7339790 32636479

[141] Shimizu K Iyoda T Sanpei A Nakazato H Okada M Ueda S. Identification of TCR repertoires in functionally competent cytotoxic T cells cross-reactive to SARS-CoV-2. Commun Biol. (2021) 4:1365. doi: 10.1038/s42003-021-02885-6 34857854 PMC8640030

[142] Olafsdottir TA Bjarnadottir K Norddahl GL Halldorsson GH Melsted P Gunnarsdottir K. HLA alleles, disease severity, and age associate with T-cell responses following infection with SARS-CoV-2. Commun Biol. (2022) 5:914. doi: 10.1038/s42003-022-03893-w 36068292 PMC9446630

[143] Kared H Redd AD Bloch EM Bonny TS Sumatoh H Kairi F. SARS-CoV-2-specific CD8+ T cell responses in convalescent COVID-19 individuals. J Clin Invest. (2021) 131:e145476. doi: 10.1172/JCI145476 33427749 PMC7919723

[144] Buckley PR Lee CH Pereira Pinho M Ottakandathil Babu R Woo J Antanaviciute A. HLA-dependent variation in SARS-CoV-2 CD8+ T cell cross-reactivity with human coronaviruses. Immunology. (2022) 166:78–103. doi: 10.1111/imm.v166.1 35143694 PMC9111820

[145] Peng Y Dong D Penkava F Mentzer AJ Yao X. An immunodominant NP105–113-B* 07: 02 cytotoxic T cell response controls viral replication and is associated with less severe COVID-19 disease. Nat Immunol. (2022) 23:50–61. doi: 10.1038/s41590-021-01084-z 34853448 PMC8709787

[146] Stanevich OV Alekseeva EI Sergeeva M Fadeev AV Komissarova KS Ivanova AA. SARS-CoV-2 escape from cytotoxic T cells during long-term COVID-19. Nat Commun. (2023) 14:149. doi: 10.1038/s41467-022-34033-x 36627290 PMC9831376

[147] Starr TN Greaney AJ Dingens AS Bloom JD. Complete map of SARS-CoV-2 RBD mutations that escape the monoclonal antibody LY-CoV555 and its cocktail with LY-CoV016. Cell Rep Med. (2021) 2:100255–5. doi: 10.1016/j.xcrm.2021.100255 PMC8020059 33842902

[148] Wellington D Yin Z Yu Z Heilig R Davis S Fischer R. SARS-CoV-2 mutations affect antigen processing by the proteasome to alter CD8+ T cell responses. Heliyon. (2023) 9:e20076. doi: 10.1016/j.heliyon.2023.e20076 37842619 PMC10570596

[149] Deng S Xu Z Wang M Hu J Liu Z Zhu F. Structural insights into immune escape at killer T cell epitope by SARS-CoV-2 Spike Y453F variants. J Biol Chem. (2024) 300:107563. doi: 10.1016/j.jbc.2024.107563 39002680 PMC11342781

[150] Chaurasia P Nguyen THO Rowntree LC Juno JA Wheatley AK Kent SJ. Structural basis of biased T cell receptor recognition of an immunodominant HLA-A2 epitope of the SARS-CoV-2 spike protein. J Biol Chem. (2021) 297:101065. doi: 10.1016/j.jbc.2021.101065 34384783 PMC8352664

[151] Wu D Efimov GA Bogolyubova AV Pierce BG Mariuzza RA. Structural insights into protection against a SARS-CoV-2 spike variant by T cell receptor diversity. J Biol Chem. (2023) 299:103035. doi: 10.1016/j.jbc.2023.103035 36806685 PMC9934920

[152] Del Val M Schlicht H-J Ruppert T Reddehase MJ Koszinowski UH. Efficient processing of an antigenic sequence for presentation by MHC class I molecules depends on its neighboring residues in the protein. Cell. (1991) 66:1145–53. doi: 10.1016/0092-8674(91)90037-Y 1913805

[153] Draenert R Gall S Pfafferott K Leslie A Chetty P Brander C. Immune selection for altered antigen processing leads to cytotoxic T lymphocyte escape in chronic HIV-1 infection. J Exp Med. (2004) 199:905–15. doi: 10.1084/jem.20031982 PMC2211885 15067030

[154] Ranasinghe SRF Kramer HB Wright C Kessler BM di Gleria K Zhang Y. The antiviral efficacy of HIV-specific CD8+ T-cells to a conserved epitope is heavily dependent on the infecting HIV-1 isolate. PloS Pathog. (2011) 7:e1001341. doi: 10.1371/journal.ppat.1001341 21589893 PMC3093356

